# Design and Assessment of Biodegradable Macroporous Cryogels as Advanced Tissue Engineering and Drug Carrying Materials

**DOI:** 10.3390/gels7030079

**Published:** 2021-06-28

**Authors:** Irina N. Savina, Mohamed Zoughaib, Abdulla A. Yergeshov

**Affiliations:** 1 School of Pharmacy and Biomolecular Sciences, University of Brighton, Huxley Building, Lewes Road, Brighton BN2 4GJ, UK; 2 Institute of Fundamental Medicine and Biology, Kazan (Volga Region) Federal University, 18 Kremlyovskaya St., 420008 Kazan, Russia; mohamadzougheib@gmail.com (M.Z.); abdulla.ergeshov@mail.ru (A.A.Y.)

**Keywords:** macroporous hydrogels, cryogels, biodegradability, natural and synthetic polymers, tissue engineering, biomaterials, drug delivery

## Abstract

Cryogels obtained by the cryotropic gelation process are macroporous hydrogels with a well-developed system of interconnected pores and shape memory. There have been significant recent advancements in our understanding of the cryotropic gelation process, and in the relationship between components, their structure and the application of the cryogels obtained. As cryogels are one of the most promising hydrogel-based biomaterials, and this field has been advancing rapidly, this review focuses on the design of biodegradable cryogels as advanced biomaterials for drug delivery and tissue engineering. The selection of a biodegradable polymer is key to the development of modern biomaterials that mimic the biological environment and the properties of artificial tissue, and are at the same time capable of being safely degraded/metabolized without any side effects. The range of biodegradable polymers utilized for cryogel formation is overviewed, including biopolymers, synthetic polymers, polymer blends, and composites. The paper discusses a cryotropic gelation method as a tool for synthesis of hydrogel materials with large, interconnected pores and mechanical, physical, chemical and biological properties, adapted for targeted biomedical applications. The effect of the composition, cross-linker, freezing conditions, and the nature of the polymer on the morphology, mechanical properties and biodegradation of cryogels is discussed. The biodegradation of cryogels and its dependence on their production and composition is overviewed. Selected representative biomedical applications demonstrate how cryogel-based materials have been used in drug delivery, tissue engineering, regenerative medicine, cancer research, and sensing.

## 1. Introduction

Hydrogels are physically or chemically cross-linked hydrophilic polymer networks, which are capable of retaining large amounts of water without dissolving. Owing to these properties and their ability to mimic the biological environment, they have been widely used in the area of tissue engineering, regenerative medicine and drug delivery. When hydrogels are used for biomedical applications, the choice of polymers is limited as they must be non-toxic and non-immunogenic, sustain sterilization, and ensure appropriate In Vivo stability or biodegradation rates depending on the requirements. In addition, to support cell attachment and growth, the hydrogel scaffolds must mimic the extracellular matrix (ECM). In most cases, the scaffold structure should be porous with a high degree of pore interconnectivity in order to support cell migration, proliferation and metabolic activity, and provide sufficient mechanical strength and stability in the body. 

Conventional techniques for making 3D porous scaffolds include solvent casting particulate leaching, gas foaming, phase separation, freeze-drying, and electrospinning [1]. Over the past 20 years, we have seen a significant increase in interest in the use of cryotropic gelation as a method of preparation of 3D porous hydrogel scaffolds [2,3,4,5]. Cryotropic gelation relies on solvent freezing to create pores in hydrogel material [6]. This is somewhat similar to freeze-drying, where a gel precursor solution or hydrogel is frozen to form a porous material and then lyophilized. No drying is carried out after cryotropic gelation and after thawing a stable macroporous hydrogel forms. A cryogel forms as a result of physical or chemical cross-linking that occurs in a sample when stored under frozen conditions at −12 °C to −20 °C. The cryotropic gelation method has been shown to be beneficial when compared to other methods that rely on physico-chemical interruptions in the hydrogel solution achieved by using porogens such as salt, sugar, silica and others, that should be removed after gelation. The careful removal of these pore-forming units is necessary to prevent them from having adverse effects on cells, but this step is hard to achieve and may cause chemical contamination of the material in addition to an inadequate interconnection of the pores [5]. Cryotropic gelation, on the other hand, has proved to be a versatile, robust and effective technique for designing porous biomaterials [5,7,8,9]. 

Although the scaffolds can be made from a non-biodegradable polymer, their use is limited, as their long presence inside the body can cause a foreign body reaction, necessitating scaffold replacement. Thus, the selection of suitable biodegradable polymers is a key consideration in the development of tissue engineering scaffolds and drug carrying materials. The choice of biodegradable polymers is determined by the need for mechanical properties and biological characteristics that can mimic the original biological environment. The performance of the scaffold also depends on the biodegradation mechanism, the rate of scaffold resorption, and the products released into the host site during this process. Although the products of resorption may be non-toxic, their release can alter the local cell environment and have a negative impact on the entire regeneration process. Thus, understanding biodegradation as well as the ability to manage it is very important when developing biomaterials. There are various ways to control the biodegradation of biomaterials. Biodegradation depends on the polymer and local environment, porosity, polymer concentration, additives, the nature of the cross-linking agent and the degree of crosslinking, which can be controlled during fabrication of the scaffold. Although cryogels have been extensively reviewed, their biodegradation, especially with regard to their production and composition, has not been well covered in previous work.

In this review, the cryotropic gelation of biodegradable polymers is discussed with a particular focus on the effect of cryotropic gelation parameters, and on the nature of polymers and their composition on the morphology and biodegradability of cryogels. Cryogels made of synthetic and natural biodegradable polymers and their composites are discussed with examples of their applications in the biomedical field. Biodegradation methods are overviewed with a focus on the effect of cryogel composition, structure, cross-linking and other conditions. Finally, examples of cryogel application in the biomedical field are presented.

## 2. Cryotropic Gelation as a Tool for the Preparation of Macroporous Scaffolds

**Figure 1:**
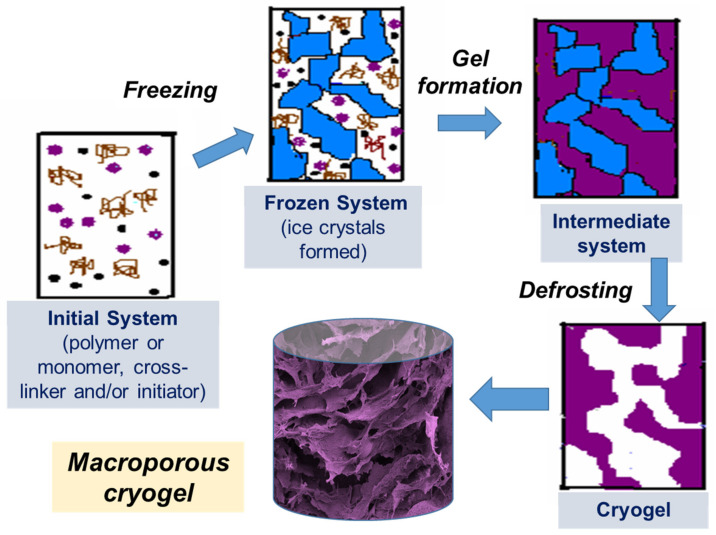
Scheme of preparation of cryogels: (i) initial system (the gel precursor solution is cooled down to a temperature below freezing point); (ii) frozen system (the ice crystals formed and solutes are concentrated in non-frozen zones); (iii) intermediate system (gel is formed in non-frozen liquid around ice crystals); (iv) cryogel is formed upon defrosting.

Cryotropic gelation is the formation of a hydrogel under semi-frozen conditions, when most of the solvent crystallizes, and gelation occurs in small non-frozen areas around the solvent crystals (Figure 1) [2,6,10]. The solvent crystals perform as a template for the pores, and after melting, they leave large voids filled with liquid solvent [6]. The most common solvent used is water, but other solvents or solvent mixtures with freezing points reasonably close to zero can be used, such as dimethyl sulfoxide (DMSO), benzene or cyclohexane [11,12]. Water is considered to be the best solvent for the formation of biocompatible cryogels for biomedical applications. Gelation can occur through different mechanisms, usually through the formation of covalent bonds, or physical interactions (ionic bonding, hydrogen bonding and others) [13]. Synthesis by radical polymerization is the most commonly used method of preparing polymer cryogels. Acrylate derivatives are mostly used as gel-forming units together with an ammonium persulphate and tetramethylethylenediamine (APS/TEMED) initiating system in these reactions. Cryogels based on natural polymers can be prepared by cross-linking intact or pre-modified polymers utilizing the amino, carboxyl, hydroxyl and other functional groups. Variation of the cryotropic gelation parameters, such as the freezing temperature, cooling rate, and the presence of the ions or other solutes, as well as the polymer and solvent content, allows the tuning of cryogel properties for a specific application.

### 2.1. Cross-Linking

Cross-linking is one of the major factors that affect the mechanical and biological properties of cryogel scaffolds and their biodegradability. The types of cross-linking are summarized in Table 1. Although cryogel scaffolds can be readily formed through physical cross-linking—for example, involving ionic interaction and hydrogen bonding—they are generally reversible, and the resultant porosity and mechanical properties will not always be satisfactory for all applications. Poly(vinyl alcohol) (PVA) is one of the most studied synthetic polymers that can be physically cross-linked to obtain cryogels [2,14]. PVA cryogels are formed as a result of the formation of microcrystalline zones and are thermally reversible. The pore size of PVA cryogels is less than 10 µm. They have been used in a number of biotechnological applications for the immobilization of enzymes [15] and cells [16], as well as in tissue engineering, mainly for mimicking bone and cartilage tissues [17,18]. 

An example of non-covalent ionic-type cross-linking is the preparation of ionic cryogels as a result of the growth of metal-polymer-coordinated complexes and electrostatic interactions between oppositely charged groups of chitosan and metal ions such as PdCl_4_^2−^, PtBr_4_ ^2−^ and PtCl_4_^2−^ at sub-zero temperatures [40,41]. Alginate-based cryogel can be obtained by introducing ionic links between anionic polysaccharide macromers with divalent cations such as Ca^2+^ [19]. 

Self-assembly of peptides is another example of the formation of non-covalent cross-linked cryogels. Peptide cryogels were obtained by pH-sensitive self-assembly of fluorenyl-9-methoxycarbonyl (Fmoc)-diphenylalanine (Phe-Phe) into fibers in an apparently frozen system [42]. Fmoc-Phe-Phe-derived cryogels were noticeably less mechanically strong than equivalent hydrogels prepared from the same concentration of Fmoc-Phe-Phe at ambient temperature. This could be due to the effect of cryostructuration: pre-concentration of Fmoc-Phe-Phe at low temperatures leading to much higher concentrations of Fmoc-Phe-Phe compared to the bulk concentration in the hydrogel precursor solution, which may affect the formation of the fibers. In addition, the formation of large pores creates a more heterogeneous structure in cryogels than in hydrogels, which can reduce the mechanical stability of the material [42]. Usually, cryogels obtained by radical polymerization or covalent cross-linking are mechanically stronger than hydrogels obtained from the same gel precursor solution at room temperature. In the case of Fmoc-Phe-Phe-derived cryogels, the 3D structure was supported by weaker non-covalent interactions, which may explain why cryogels were mechanically less strong.

Covalent cross-linkers are selected based on the chemistry of the polymer used. Bifunctional acrylate derivatives, such as *N*,*N*′-methylenebisacrylamide (MBA), poly(ethylene glycol) diacrylate (PEGDA) and others have been used to provide additional connections between formed polymer chains during radical polymerization. A range of different cross-linkers are used for cross-linking biological polymers. 1-ethyl-3-(3-dimethylaminopropyl)carbodiimide (EDC), alone [43,44] or in combination with N-hydroxysuccinimide (NHS) [45,46], is an important water-soluble activator for carboxyl and phosphate groups of biopolymers that makes them reactive, respectively, toward amino and hydroxyl groups. EDC/NHS generates peptide-like bonds and is well used as a potentially biocompatible cross-linker in the biomaterial fields. EDC is limited to cross-link molecules that are directly adjacent to each other, whereas glutaraldehyde (GA) provides more flexible cross-linking with longer linkage between molecules. 

GA has been widely used as a cross-linking agent for collagen, gelatin, chitosan and other polymers due to its low cost, high solubility and stability in aqueous solutions [47,48]. However, the excessive incorporation of GA into scaffolds can have implications for biocompatibility. Some adverse effects of GA modifier, such as the inhibition of cell proliferation, have been reported, and it was suggested that the lowest concentration that does not compromise the mechanical properties of the cryogel should be used [49]. Additionally, to improve biocompatibility of the scaffolds cross-linked with GA, residual aldehyde groups and formed Schiff’s base in the cryogel have to be reduced by sodium borohydride [25,50]. The removal of toxic sodium borohydride itself requires extensive washing of the material, which can be time-consuming and expensive. Alternatively, a hydrophilic polysaccharide dextran pre-modified with aldehyde groups was used as a cross-linker for gelatin and chitosan [51,52]. The viability of mouse embryo NIH 3T3 fibroblasts was considerably higher (∼80%) on modified dextran cross-linked scaffolds than on scaffold cross-linked with GA (40%) [51], which indicates a lower toxicity of the modified dextran cross-linked scaffolds. 

Genipin, a natural product extracted from the gardenia fruit, has been utilized as a biocompatible cross-linker to obtain hyaluronic acid (HA) based cryogels [53]. The effect of GA, genipin, and EDC/NHS on the formation of carrageenan-alginate scaffolds by freeze-drying was analyzed [45]. Scaffolds produced using EDC/NHS cross-linking resulted in better cellular response and higher metabolic activity, as well as demonstrating biocompatibility properties, and being porous and physically and mechanically stable. Thus, even where genipin has been proposed as a non-toxic cross-linker, the final choice of cross-linker must be determined by the application and properties of the scaffold, and an alternative cross-linkers must be taken into consideration in order to achieve best results. Another limitation of genipin is that cross-linking produces a material that has a strong blue color, which is not always desirable.

Kirsebom et al. studied the cryotropic gelation of gelatin and casein catalyzed by transglutaminase (TGase) enzyme. TGase catalyzes the linkage of the γ-carboxyamide group of glutamine and the ε-amino group of lysine [54]. The enzymatic cross-linking under partly frozen conditions was a slow process, requiring at least 14 days to form gels that were sufficiently stable to handle. The gels, however, were not formed at room temperature, indicating the role of freezing in the gelation process. The mechanical properties of gelatin and casein cryogels improved with increasing protein content [54]. There is some potential advantage to enzymatic cross-linking, such as mild reaction conditions and no need to use other chemicals as cross-linking agent. However, the enzyme activity under cryogenic conditions is low and cryogel formation takes a long time.

The use of external energy sources, such as UV radiation, is another alternative in the synthesis of cryogels which does not use chemical initiators. Cryogels from mixed semi-dilute solutions of 2-hydroxyethylcellulose and chitosan have been obtained using UV radiation with H_2_O_2_ as a photoinitiator [55]. The advantages of this process are the relatively short time required for cryogel formation and the fact that no additional purification is needed. Unfortunately, this method is limited by the low penetration depth of photons. Another approach to cross-linking was used in [35,36,37], where polymerization of acrylated dextran and hyaluronan was initiated by accelerated electrons (an E-beam). This method uses no toxic initiators, and results in sterile, elastic scaffolds with a controlled pore size, excellent swelling and low flow-resistance properties [37]. The reaction time was short—less than 10 min—and with double-sided irradiation, cryogels up to 7 cm thick could be obtained. Some degradation occurred under irradiation along with cross-linking, and the degradation products were washed out, resulting in 80% of the hyaluronan being incorporated into the scaffold [35]. Even though this is a simple cross-linking process, it requires special electron beam equipment and the modification of polysaccharides with acrylate groups, followed by extensive purification before polymerization. Another disadvantage is the limitation of the cryogel size that can be produced and the impossibility of using it to obtain larger samples.

### 2.2. Effect of Composition

The composition—that is, the nature of the polymers used, their molecular weight and concentration—have an effect on the final cryogel morphology, mechanical properties and degradation. The porosity and mechanical properties of a HA cryogel cross-linked with ethylene glycol diglycidyl ether (EGDE) has been shown to depend on the HA and cross-linker concentration [56]. Increasing HA concentration decreased cryogel pore size, reduced swelling properties, and reinforced mechanical properties. On the other hand, decreasing cross-linker concentration, at a constant HA concentration, increased the pore size and swelling capacity but provided less rigidity [56]. The composition of gelatin/HA cryogels had an effect on the compressive modulus and other important mechanical characteristics of the cryogels [57]. Larger amounts of gelatin in the composite cryogel increased its Young’s modulus (or stiffness) and toughness due to the formation of additional EDC-mediated cross-links between gelatin and HA molecules, which contributed to the resistance of the struts to bending under compression. A more cross-linked material also showed diminished ultimate strain. However, increasing the gelatin concentration beyond 10% was found to offer an excessive cross-linking rate at the cross-linking temperature (−20 °C) and cross-linker concentration used. This resulted in completion of the gelation process in the polymer solution before porogens (ice crystals) were formed. The cryogels formed under these conditions were very brittle and had low porosity and inferior mechanical strength [57]. These examples show that the composition of the cryogel must be optimized for each specific application with respect to the nature and concentration of the polymer, as well as the cross-linking mechanism.

### 2.3. Effect of Freezing Conditions

The freezing temperature affects the pore size and wall thickness of cryogels [26,56]. The highest freezing temperature and the slowest cooling rate produces the biggest pores in cryogels and lower temperature and faster freezing rates are required to produce the smallest pores. The reason for this is the different rates of growth of solvent crystals. Bigger solvent crystals form at slower cooling rates, and this creates bigger pores. At lower temperatures, more solvent will freeze, creating less space between the solvent crystals, where the gel precursors will concentrate forming denser polymer walls. At lower temperatures, the rate of nucleation is higher, which leads to the formation of more crystals of smaller size, thus creating smaller pores in the final cryogel material [26].

In general, the solvent crystals must form before gelation has finished. When cryogels are synthesized by radical polymerization, a lower concentration of initiator is usually used to slow down the polymerization rate and produce cryogels with large pores [58]. If the gelation occurs faster than the ice crystal formation, large, interconnected pores will not form and the cryogel will have a morphology similar to a hydrogel obtained at room temperature. A gel formed under these conditions will have less porosity, less mechanical strength, and will be brittle in nature [57]. Hixon et al. compared cryogel scaffolds with the conventional hydrogel-based scaffolds of three different materials (chitosan-gelatin, N-vinyl-2-pyrrolidone, and silk fibroin (SF)) to identify the optimal material and form of scaffold for use as a graft substitute in bone regeneration [8]. Cryogels proved superior to hydrogels in terms of swelling potential, porosity and mechanical properties, regardless of the polymer used [8]. The microstructure of the cryogels depended on the kinetics of the gelation and nucleation and could be controlled, as mentioned above, by temperature as well as by the concentrations of the initiator or cross-linker [58]. 

**Figure 2:**
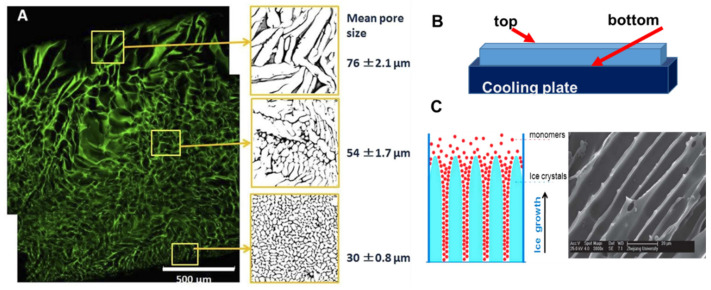
Anisotropic structure in cryogels: (**A**) gelatin cryogel frozen in a petri dish, (**B**) scheme of set up for unidirectional freezing, and (**C**) the scheme of ice-crystals grows and scanning electron microscopy image of PEG-based cryogel. (Figure (**A**) is adapted from [6] and used under a Creative Commons Attribution 4.0 International License and (**C**) is adapted from [59] with permission from The Royal Society of Chemistry.

A heterogeneous structure of the cryogels forms under unidirectional freezing. This was observed in the preparation of sheets of cryogels, where the pore size varied from top to bottom. The cryogel had smaller pores at the bottom (in contact with the freezing plate) and the pore size gradually increased towards the top (Figure 2A) [27]. This anisotropic morphology can be beneficial for wound healing because it creates a special environment for cells and controls their migration. When material is applied with smaller pores at the top and larger pores at the wound bed, it stimulates cell migration from the wound into the material. An additional benefit of placing larger pores of the material on the wound bed is increased flexibility to adjust to the roughness and curvature of the wound surface [27].

Many natural tissues, such as those in nerves, cartilage, ligaments, tendons, and the spinal cord, have oriented structures. The morphology of tissue scaffolds should mimic these natural tissues, and cryogels with an aligned porous structure are preferable for better physiological and mechanical function of the tissue scaffold. Unidirectional freezing has been used to create aligned porous structures in both synthetic and natural polymer cryogels [59,60]. During unidirectional freezing, ice crystals form and grow in one direction, forming an aligned porous structure after melting (Figure 2C). Poly(ethylene glycol) cryogels were prepared using PEGDA of various molecular weights: 200, 700 and 2000 Da as a precursor [59]. Cryogels with a smaller pore diameter were obtained using lower molecular weight 200 Da, PEGDA-200, while larger pores were observed when PEGDA with a molecular weight of 2000 Da was used (PEGDA-2000). The smaller pores of the PEGDA-200 cryogel were observed because PEGDA-200 exhibited a marked tendency to adsorb on the surface of the ice crystal during freezing, inhibiting crystal growth. Smaller ice crystals resulted in smaller pore diameters [59]. Freezing temperature was an important parameter for unidirectional freezing because it influenced the rate of formation, size and orientation of solvent crystals. Using a higher temperature leads to the formation of cryogels with large pore diameters. In the work by Wu et al., frozen acetic ether, frozen ethyl alcohol and liquid nitrogen were used to achieve freezing temperatures of −80 °C, −110 °C and −196 °C, respectively. The resulting cryogels had anisotropic compressive strength in accordance with the directions of the pores, and the diameter of the microtubule channels could be controlled in the range 10 to 50 μm by adjusting the molecular weight of the PEGDA and the freezing temperature [59]. Using the same approach, a chitosan-gelatin cryogel with aligned porous structure was prepared inside a polyurethane tube for peripheral nerve regeneration [60].

### 2.4. Bacterial Cell Based Cryogels

**Figure 3:**
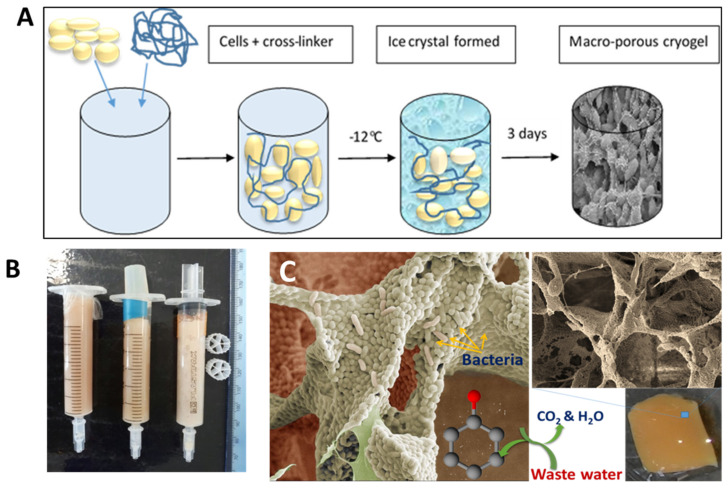
(**A**) Scheme of bacterial cryogel preparation, (**B**) bacterial cryogels prepared in syringes, and (**C**) SEM images of bacterial cryogel and scheme of phenol compound degradation. Figures are adapted from [62] with permission from the Royal Society of Chemistry.

Cryogels can be made from bacterial cells by cross-linking them with a biocompatible cross-linker [61,62,63]. This approach uses living bacterial cells as the main building blocks for cryogels. Bacterial cryogels were prepared in a one-step process with a low percentage of polymer cross-linker, which formed a three-dimensional porous structure with a well-developed system of interconnected pores [62] (Figure 3). A range of the cryogels were made from whole cells of *Escherichia coli* [61], *Pseudomonas mendocina*, *Rhodoccocus koreensis* [62] and *Acinetobacter radioresistens* [63] and their combination [62]. Various macromolecular cross-linking agents were used, such as oxidized dextran, PVA and polyethyleneimine (PEI), the latter two being activated by GA. The composition of cross-linker agents was optimized so that a high percentage of cells remained viable (50 to 80%) and retained their metabolic activity [62,63]. As can be seen from the SEM image, the walls of the cryogels were mainly composed of whole cells with a very low percentage of polymer, around 1 to 2% (Figure 3C). Bioreactors, based on bacterial cryogels, were effective for the degradation of phenolic compounds [62,63]. The authors believe that the aforementioned approaches can also be used to design cryogels containing some other bacteria with beneficial medical effects or potentially extended to human cells, which nevertheless will require sophisticated conditions to maintain cell viability and activity under cryogenic conditions. 

### 2.5. 3D Printing of Cryogels

**Figure 4:**
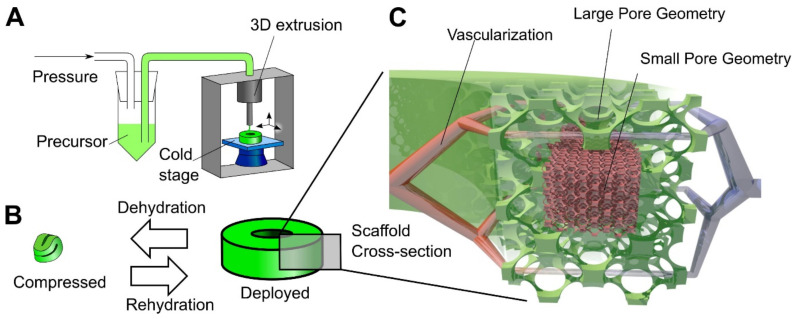
Principle of 3D cryogel printing. (**A**) Illustration of 3D printing of cryogels. A viscous precursor solution was printed through a modified commercial printer onto a freezing cold stage. The liquid froze upon stage contact, leading to local self-organization of ice crystals and polymer. After curing at –20 °C and thawing, a cryogel was obtained in the desired shape (in this example, a torus). (**B**) Illustration of the 4D shape change. By withdrawing fluid, the cryogel was highly dehydrated and compressed; in addition, surface tension drove surface-minimizing folding. Upon rehydration, the cryogel returned to its original shape and volume via shape memory. The switch between compressed and deployed cryogel state was reversible, could be repeated many times, and was used for minimally invasive delivery. (**C**) Illustration of the hierarchical scaffold organization. Local pore size variation was used to preferentially drive in vitro cellular organization and In Vivo vascularization to areas with large pores. Reprinted from Acta Biomaterialia, Vol 76, Beduer, A.; Piacentini, N.; Aeberli, L.; Da Silva, A.; Verheyen, C.A.; Bonini, F.; Rochat, A.; Filippova, A.; Serex, L.; Renaud, P.; et al. Additive manufacturing of hierarchical injectable scaffolds for tissue engineering. Copyright 2018 [64] with permission from Elsevier.

Another step towards creating a complex tissue design with better control over porosity and material heterogeneity is 3D printing. The cryogel 3D printing process has been designed to provide better control over porosity in cryogel samples [64,65]. Carboxymethylcellulose was used as a structural material with cross-linking chemistry of adipic dihydrazide. A multilayer 3D printing of cryogels was performed by controlling the temperature of the dispensed solution, which allows to change the local pore size on demand (Figure 4) [64]. It was possible to control the cell seeding density in vitro, as well as tissue infiltration and vascularization In Vivo by precisely controlling the local geometry of the scaffold in a larger 3D structure [64]. Another example of 3D printing involved the preparation of self-healing, oxime cryogels by cross-linking a poly(n-hydroxyethyl acrylamide-co-methyl vinyl ketone) with a bi-functional hydroxylamine [66]. 

## 3. Biodegradable Cryogels

Biodegradable cryogels have been prepared from natural polymers (biopolymers) and synthetic polymers with biodegradable/bioresponsive bonds containing bio-resembling or artificial units. The cryogels could be composed of one type of polymer or a combination of different materials. Polymers from natural origins (plants or animals) attract considerable attention for their intrinsic biocompatibility and potential ability to mimic ECM, favoring cell-matrix interactions for tissue-engineering and regeneration. Several synthetic polymers, such as poly(ethylene glycol) (PEG) [58,67], poly(L-lactic acid) (PLLA) [68] and PVA, have also been utilized for cryogel formation [18]. The mechanical properties and degradation of the synthetic polymers can be easily adjusted by changing the molecular weight, composition and types of bonds. The main drawbacks of using these polymers as scaffolds are the lack of bio-adhesive and cell-stimulating properties. This can be compensated for by modifying the scaffold with bioactive molecules or by combining with natural polymers to form composites, as discussed below. 

### 3.1. Natural Polymers 

The properties of natural polymers and examples of their use for cryogel formation are summarized in Table 2. Below, we briefly outline the most common biopolymers used for making cryogels.

#### 3.1.1. Proteins and Peptides

##### Collagen

Collagen is the main component of ECM and the most abundant protein in mammalian tissues. Collagen is a fibrous structural protein consisting of a right-handed triple helix of three parallel left-handed polypeptide strands (polyproline II-type helical conformation). It is soluble in acidic aqueous solutions and naturally degraded by secreted matrix metalloproteinases and serine proteases. Among the several types of collagen, the most abundant in the body are types I, II, III and IV, which form the major components of connective tissue proteins in skin, tendon, bone, cornea, ligament, intestine, and blood vessels. Due to this, collagen is often a favorable material for the development of a tissue scaffold that mimics the ECM environment and promotes cell proliferation and differentiation. A collagen-based cryogel was prepared by cross-linking with EDC/NHS [103,104]. Coating with polydopamine increased the viability (by 52%) and proliferation (by 33%) of mesenchymal stem cells in dopamine-modified collagen cryogels compared to non-modified ones [103]. Pure collagen is often used to form a scaffold, not alone but in combination with various materials, as discussed in other sections [90,91,112].

##### Gelatin

Gelatin is a denatured collagen analogue and can be isolated from animal skin, bone and cartilage tissues. The isolation involves melting and partial hydrolysis of collagen triple helix to produce separate random coil polypeptides called gelatin. Gelatin is composed primarily of repeating amino acids: glycine, proline, and hydroxyproline. It has free carboxyl and amino groups in its backbone and is positively charged in aqueous solution. It is also known that gelatin has an arginine-glycine-aspartic acid (RGD) sequence that promotes cell attachment and proliferation [43]. Gelatin has several advantages over collagen, which relate to its low cost, stability, aqueous solubility, purity and the loss of some of the immunogenic sites of native collagen. It is one of the most widely used materials for manufacturing cryogels. Various types of gelatin are used, including bovine gelatin [107], porcine gelatin [106] and piscine gelatin [27]. Piscine gelatin has a different amino acid content and a lower melting point [113], which results in the formation of cryogels of different mechanical strength and degree of swelling compared to porcine or bovine cryogels. It is also believed to have a low potential for the transmission of infectious agents such as viruses and prions, and is therefore widely used to form cryogels; in particular, cold fish gelatin is mainly used [25,27,28,50]. Gelatin-based cryogels are commonly produced by cross-linking with GA [27,28,50,107]. They can also be made by radical polymerization of methacrylated gelatin molecules in the presence of APS/TEMED [114]. The resulting cryogels demonstrate high biocompatibility revealed on 3T3 fibroblasts, which were characterized by enhanced attachment and spindle-like morphology. They also provided the potential for protein encapsulation and localized release upon enzymatic degradation by collagenases or mammalian MMP-3 and 9 [114]. Gelatin and gelatin/HA cryogels cross-linked with EDC were obtained for mesothelial cell culture [106]. The addition of HA in cryogel resulted in faster biodegradation by collagenase and a higher elastic modulus compared to the gelatin cryogel. The addition of HA to the cryogel adversely affects the behavior of mesothelial cells during 3D cell culture. Cells cultured in gelatin-HA scaffolds showed changes in their morphology, cytoskeleton arrangement, and proliferation rate. For gelatin-HA, compared to the gelatin cryogel, the downregulation of the mesothelium-specific maker gene, ICAM1, together with reduced production of the key mesothelium proteins, E-cadherin and calretinin, was noted [106].

##### Platelet lysate (PL) 

The use of autologous components as biomaterials is highly regarded as it can eliminate both tissue rejection and potential transmission of animal diseases and often favors regeneration. There are limited supplies of autologous tissues in the body and blood-derived products are an important focus of attention [108]. Platelet lysate (PL) is a blood-derived autologous product obtained by the lysis of concentrated platelets. PL contains large amounts of growth factors and cytokines responsible for the regulation of angiogenesis and wound healing, and has been considered for the preparation of wound healing materials [108]. An oxidized dextran has been used to crosslink PL components and to form composite cryogels. These cryogels supported cell survival and chondrogenic differentiation of human adipose stem cells (hASC) in vitro in the presence of chondroinductive factors. PL cryogels were found to be biodegradable within 90 to 240 days after subcutaneous implantation in rats [108]. In another work, PL was cross-linked by aldehyde functionalized cellulose nanocrystals. Cellulose nanocrystals significantly reinforce the low strength of the PL-based matrix due to the covalent cross-linking of its amine groups and enable the formation of an elastic interconnected porous network [77]. The proposed PL cryogel has been tested as an alternative off-the-shelf hemostatic and antibacterial biomaterial with the potential to deliver therapeutically relevant proteins In Situ.

##### Silk Fibroin

Silk, obtained from silkworms, consists mainly of the proteins sericin and fibroin and has recently been studied in many applications of tissue engineering and regenerative medicine. Silk fibroin-based cryogels were synthesized by freeze-thawing [8,115] and using butanediol diglycidyl ether as a cross-linker [109]. The addition of ethylene glycol diglycidyl ether into the cryotropic gelation system triggers the conformational transition of fibroin from a random coil to the beta-sheet structure and, consequently, the gelation of fibroin during the freeze-thawing process [116]. The cryogel scaffolds produced were very strong, with Young’s modulus between 50 and 126 MPa [109,116], and sustained around 90% compression under stresses of 87 to 240 MPa, which makes them a good material for bone tissue engineering [109]. The mechanical properties improved considerably as a result of multiple-networking and increased fibroin content [109].

For the manufacture of cryogels, proteins such as albumin, casein, fibrinogen, elastin and others were also used, information on which is presented in Table 2.

#### 3.1.2. Polysaccharides

##### Agarose

Agarose is a linear polysaccharide, a copolymer of (1→3)-linked β-D-galactopyranose and (1→4)-linked 3,6-anhydro-α-L-galactopyranose, and has some sulfate groups. It is a constituent of agar isolated from the red seaweed of the *Rhodophyceae phylum* [117]. It is water-soluble at temperatures above 65 °C and can gel in a range of temperatures from 17 °C to 40 °C below the gel-melting temperature 90 °C, depending upon the molecular weight and chemical modification of side groups. Agarose-based cryogels were prepared by a two-step freezing procedure (freezing at −30 °C followed by incubation at a higher sub-zero temperature) and subsequent thawing [22]. These cryogels, formed as a result of physical interactions, intramolecular and intermolecular hydrogen bonding, are thermo-reversible and can be melted by heating above the gel fusion temperature [22]. They have been used as scaffolds for culturing insulin-producing cell aggregates. Due to its excellent biocompatibility and physicochemical properties, agarose is commonly used in combination with other biopolymers to make cryogels suitable for tissue engineering applications. Agarose has been added to cryogel composition together with gelatin, alginate and chitosan to improve the mechanical properties of cryogels [118,119], which is discussed in the following section. 

##### Alginates

Composed of β-D-mannuronate and α-L-guluronate monomers, this copolymer is often ionically cross-linked with cations, such as Ca^2+^. Alginate hydrogel beads cross-linked with Ca^2+^ were subjected to three freeze-thaw processes to obtain cryogels [19]. These cryogels were tested for the release of quercetin, the bioactive flavonoid, encapsulated in the beads. The mechanical properties of alginate gels obtained by ionic cross-linking with Ca^2+^ are difficult to control. To overcome this, Boulais et al. performed a covalent cross-linking with chemical agents, the adipic acid dihydrazide (AAD), a symmetrical molecule with primary amines at each end, and EDC to obtain alginate cryogels [74]. The carboxylic group of alginate monomers was first activated by EDC, followed by reaction with AAD. This leads to the creation of amide bonds by the coupling of the amino groups of AAD to the activated carboxyl groups of alginate monomers, resulting in the desired product alginate amide. An increase in the amount of cross-linking agents significantly improves the mechanical properties, but at the expense of the loss of porosity. It was found that the optimal parameters for the preparation of the cryogel are a concentration of alginate of 1% and 2% (*w*/*v*), and mole ratios AAD:EDC of 1:1 and 1:2, respectively. These cryogels have a macroporous structure, and their mechanical properties correspond to those of a healthy liver [74].

In other studies, cryotropic gelation of methacrylated alginate (MA-alginate) by free-radical polymerization was performed, first to create a porous alginate, which was then further cross-linked by calcium ions to enhance the toughness and injectability of the cryogel [71,72]. The tough materials obtained have been used as injectable platforms for cancer vaccination. Transplantation of irradiated breast cancer cells (as a tumor antigen) seeded into a cryogel of cross-linked MA-alginate, preloaded with macrophage colony-stimulating factor (GM-CSF) and cytosine-phosphodiester-guanine oligonucleotide (CpG-ODN) resulted in strong antigen-specific cellular and humoral responses In Vivo, respectively. The efficient production of protective anti-tumor antibodies and the activation of dendritic cells was observed. This provides a powerful prophylactic effect against a model of breast cancer in mice [72]. For combined chemo-immunotherapy purposes, injectable alginate cryogels loaded with GM-CSF and CpG-ODN were used in combination with spermine-modified acetylated dextran nanoparticles loaded with Nutlin-3a (potent p53 activator). The nanoparticles were successfully incorporated within the cryogel and further released over time. This efficiently enhanced their accumulation in tumors following peritumoral injection in mice. The delivered Nutlin-3a was toxic to the tumor cells and induced immunogenic cell death. The latter was greatly supported by the activation of antigen presenting cells recruited by GM-CSF and CpG-ODN released from the cryogel [71]. 

##### Carrageenan

Carrageenan is a linear sulfated polysaccharide isolated primarily from red seaweeds. It is regularly used in the food industry for its gelling and thickening properties. Carrageenan/gelatin cryogels were obtained by cross-linking with GA or EDC/NHS [46]. The properties of the cryogels depended on the composition and cross-linker concentration. In other work, hydrogels of carrageenan/cellulose nanofibrils as carriers for antimicrobial alpha-aminophosphonate derivatives were produced by cross-linking with glyoxal and using freeze-drying [76]. The addition of cellulose nanofibrils significantly strengthens the material, improving its mechanical properties. These scaffolds have been proposed as antimicrobial wound-healing materials and have been shown to be effective against *Staphylococcus aureus*. Nourmohammadi et al. developed cryogel scaffolds with different amounts of carrageenan added to the silk fibroin solution. When using an increased content of carrageenan, the average pore size and porosity increased. This was accompanied by better cellular proliferation of osteoblast-like cells (MG 63) and higher alkaline phosphatase expression, indicating the suitability of these scaffolds for bone regeneration applications.

##### Chitosan

Chitosan is a linear polysaccharide consisting of β(1–4)-linked D-glucosamine residues with a variable number of randomly located N-acetyl-glucosamine groups. It is prepared by *N*-deacetylation of chitin. It is generally insoluble in neutral conditions but easily soluble in the presence of an acid due to the protonation of the free amino groups of glucosamine. It is analogous to the glucosaminoglycans (GAG) found in the ECM of cartilage and can therefore mimic the ECM environment [51]. Chitosan has been shown to have excellent biocompatibility, biodegradability and adsorption properties and can easily be degraded by lysozyme, a naturally occurring enzyme. It is available in different degrees of deacetylation, viscosity and molecular weight, all of which affect the final cryogel properties. GA is highly reactive in acidic media and is often used for cross-linking chitosan during cryogel formation [26,41,79]. Diglycidyl ethers have been suggested as alternative cross-linkers to relatively toxic dialdehydes [31]. The efficacy of interaction between chitosan and diglycidyl ethers of glycols has been shown to depend significantly on the nature of the acid used to dissolve chitosan and the pH [31]. It was found that cryo-concentration of chitosan at −10 °C facilitates the formation of cross-links and, despite the lower degree of modification compared to gels obtained at room temperature, chitosan cryogels with Young’s moduli up to 90 kPa were obtained [31]. Chitosan is widely used in the preparation of tissue-engineered scaffolds for the regeneration of bones, cartilage and skin [26,31,41,51,79].

##### Dextran

Dextran, a water soluble bacterial-derived polysaccharide, consists mainly of α-1,6 linked D-glucopyranose residues with a few percent of α-1,2-, α-1,3-, or α-1,4-linked side chains [82]. It has been used in various applications in the food industry, biomedicine and nanomedicine. Cryogels have been prepared by radical polymerization of the methacrylated dextran in combination with other monomers [82,83]. Another approach used is electron-beam initiated cross-linking reaction that does not involve additional cross-linkers, potentially toxic initiators or leaching agents [36,120]. The morphology and mechanical properties of the obtained dextran cryogels were tailored by a simple variation of the macromonomer concentration. Ari et al. have used DVS for cross-linking non-modified dextran [84]. An increase of molecular weight of dextran and the concentration of DVS decreases the total pore volume and the swelling of cryogels. Dextran cryogels were synthesized via direct UV-induced cross-linking of non-modified high molecular weight dextran with *N*,*N*′-methylenebisacrylamide as cross-linker [38]. The dextran cryogels were assessed as carriers of the model water-soluble drug metoprolol. In other works, modified dextran has been used as a non-toxic cross-linker for other polymers [51,52,108].

##### Pectin

Pectin is an anionic, water-soluble polysaccharide constituting the cell walls of most plants. The main chain consists of α-1,4-linked D-galacturonic acid. It is usually extracted from various types of fruits via enzymatic or chemical methods, and is used in food as a gelling agent, particularly in jams and jellies, and as a stabilizer in fruit juices and milk drinks. Gelation of pectin depends on the pectin concentration, pH and presence of metal ions. Physically cross-linked pectin cryogels have been obtained by freeze-drying and adding Ca^2+^ and used to encapsulate and release theophylline [92]. Cryogels based on apple pectin (AP) and *Heracleum* pectin (HP) were obtained by ionic cryotropic gelation in the presence of Ca^2+^ ions. The AP cryogels showed a faster In Vivo degradation time and release of components compared to HP cryogels, resulting in a uniform covering of the wound surface and preventing the formation of adhesions in the abdominal cavity. Owing to their high anti-adhesive properties and biocompatibility, AP cryogels have been proposed for the development of a barrier material for use in surgery [93]. Chitosan/pectin cryogels were produced [20,80,93]. The cryogels were formed by both electrostatic polyelectrolyte interactions between pectin and chitosan and cross-linking of chitosan with GA [80]. The addition of chitosan to the composition of AP cryogels increases their mechanical strength and degradation time, but reduces their anti-adhesive activity, which was dependent on the chitosan concentration added. It is noteworthy that the presence of a low-molecular-weight chitosan in AP cryogels inhibits macrophage adhesion and does not induce the activation of the complement system, in contrast to cryogels based on HP. These observations, in addition to low toxicity and hemocompatibility, showed that AP cryogels and AP-chitosan composite cryogels are highly biocompatible with great biomedical potential [93].

#### 3.1.3. Glucosamine 

##### Chondroitin Sulfate 

Chondroitin sulfate is a water-soluble linear glycosaminoglycan (GAG) composed of a chain of alternating units of glucuronic acid and *N*-acetylgalactosamine, the latter being sulfated at O6 [121]. It is covalently linked to proteins to generate proteoglycans. Chondroitin sulfate is an important part of articular cartilage and provides much of its resistance to compression. Inclusion of chondroitin sulfate in scaffold may promote the secretion of proteoglycan and type II collagen specific for cartilage tissues [121]. Chondroitin sulfate was added to the gelatin, HA and chitosan cryogels to improve scaffold properties, cell proliferation and differentiation [94,121,122].

##### Hyaluronic Acid

Hyaluronic acid is composed of *N*-acetylglucosamine and *D*-glucuronic acid, which are linked via β-(1,3) bonds. It is one of the main components of connective tissue of skin and eye lens. The molecules of the HA carry a substantial negative charge and repulsive forces keep molecules apart, enabling the adsorption of a large amount of water about 1000 times of its own weight [43]. Several cross-linkers can be used to prepare HA hydrogels, such as DVS, GA, EDC, EGDE, butanediol diglycidyl ether (BDDE) and poly(ethylene glycol) diglycidyl ether (PEGDE) [123]. Compared to HA-based hydrogels, cryogels provided a better microenvironment for chondrocytes adhesion, proliferation and the biosynthesis of cartilage ECM glycosaminoglycans, demonstrating the potential use of these materials for cartilage tissue regeneration [98]. Likewise, HA cryogels obtained via UV photo-cross-linking of the HA methacrylate promoted the proliferation of chondrocytes with a twofold higher cell density compared to their equivalent hydrogel constructs in addition to increasing collagen II production by cells. The developed cryogels can serve as a platform for the creation of cell carriers, which emphasizes their potential for use in tissue engineering and regenerative medicine [39]. HA cryogels were prepared by using EDGE cross-linking [56,123]. The porosity, mechanical properties and swelling capacity of cryogels were controlled by variation of the pH, freezing temperature, polymer and cross-linker concentration. The local elastic properties of the polymer matrix and the viscous properties were, for the first time, characterized using multiple particle-tracking microrheology [56]. It has been shown that cryogels can be used as a stress-bearing material in biomedical applications [123].

### 3.2. Synthetic Biodegradable Polymers

The most common synthetic polymers that have been widely investigated for the fabrication of cryogels are PVA, pHEMA, PEG and PLLA [18,124,125,126]. Not all of them are fully degradable in the biological environment, and in this section, we will only give examples of biodegradable ones. It also appeared that synthetic polymers do not provide sufficient clues for cell attachment and proliferation and are therefore often combined with natural polymers or modified with biological active molecules. 

PEG-based materials are widely used in tissue engineering and regenerative medicine due to their soft tissue-like properties, good biocompatibility, and highly tunable properties. Mechanical and physical properties can be altered by changing the molecular weight, concentration and functionality of the PEG. The poly(ether) backbone of PEG is hydrolytically stable and so the traditional acrylate-derivative PEG (PEGDA) hydrogels, but undergo slow degradation In Vivo [127]. Significant degradation of PEGDA hydrogels occurred over 12 weeks In Vivo as a result of hydrolysis of the PEGDA end groups esters. PEG cryogels were synthesized by radical polymerization of PEGDA using APS/TEMED [128,129,130] or via UV radiation [112,131]. The morphology and swelling of the PEG cryogels were controlled by the freezing temperature and initiator concentration [58] and depended on the PEGDA concentration [131].

The degradation of PEG-based cryogels could be adjusted by modifying the end group chemistry, cross-linking mode and/or by using co-polymerization with other polymers. Dispinar et al. have prepared disulfide-containing degradable PEG cryogels using a radical-free conjugate addition strategy, Michael addition [132]. Low molecular weight PEG-based building blocks with amine end groups and disulfide-containing building blocks with maleimide end groups were combined to synthesize redox-responsive PEG cryogels. The reaction between a maleimide double bond and amine groups cross-links PEG-based building blocks. The cryogels were stable under physiological conditions due to the presence of disulphide bridges in the cryogel structure, but they completely dissolved into water-soluble products in the presence of glutathione, as a reducing agent in the medium. Cell viability experiments clearly showed that neither the degradation products nor the gel structure itself were toxic to the cells. 

A new class of scaffolds, dendrimer cryogels, have been prepared using the aza-Michael addition reaction between hyperbranched amine-terminated polyamidoamine (PAMAM G4) dendrimer and linear polyethylene glycol diacrylate at sub-zero temperatures [133]. A cross-linking network is formed as a result of reaction of the nucleophilic amines on the PAMAM dendrimer surface with α,β-unsaturated ester of the terminal acrylate groups in linear polyethylene glycol diacrylate in water. This is considered to be a green approach to the cryogel synthesis as no additional harmful chemicals (cross-linker, catalyst) were used for the dendrimer cryogel formation. The obtained dendrimer cryogels had pH-dependent swelling and degradation. They were stable at acidic pH but degraded rapidly at physiological pH due to self-triggered degradation. 

Biodegradable PEG-based cryogels were prepared by chemical cross-linking (EDC/sulfo-NHS chemistry) of amino terminated four-arm PEG and heparin (HEP) [95]. The injectable cryogels and carriers for the intrahepatic transplantation of allogeneic pancreatic islets has been prepared on their base [96,134]. PEG-based microcryogels were prepared as a mechanical skeleton to reinforce alginate encapsulation of MSCs and enable pre-formed alginate hydrogel to be injectable [135,136]. The cryogels had been formed in 45 × 14 microstencil array chip and had a size from 100 to 800 µm. The PEGDA-derived microcryogels exhibited good biocompatibility, macroporosity and strong mechanical elasticity, which made them a more desirable injectable delivery system compared to their equivalent hydrogel-based microparticles. 

### 3.3. Composite(Hybrid) Cryogels 

Cryogel-based scaffolds often consist of more than one component. These can be blends, mixtures of two or more polymers, or composites. Hybrid cryogels can be made using both types of polymers (natural or synthetic) and additives (e.g., nanoparticles, fibers) to obtain a material with advanced physical, chemical and biological properties. These materials can combine the beneficial properties of each component used in the preparation of hybrid cryogels. Thus, a biodegradable porous scaffold was prepared using collagen, HA and gelatin with great potential for skin regeneration due to a suitable pore size, high swelling, cytocompatibility, and increased collagen production by skin cells during culturing [44]. By combining advantages offered by gelatin and HA towards chondrocytes, gelatin/HA scaffolds with glucosamine (GlcN) have been prepared [43]. Glucosamine had been added to the composition of gelatin-HA cryogels as a signaling molecule with the ability to promote chondrogenesis. Physico-chemical properties evaluation revealed a similar pore size for the scaffolds without and with 9 and 16% of GlcN, but a higher porosity, degradation, and swelling ratio after incorporation of GlcN. The Young’s modulus, storage modulus, ultimate compressive stress, energy dissipation level, and rate of stress relaxation decreased, while the elasticity increased by increasing the GlcN content in the cryogel. This was believed to be due to the low cross-link density, when gelatin was substituted with GlcN. In vitro cell culture experiments using rabbit articular chondrocytes revealed that GlcN incorporation affected the cell proliferation, morphology, and maintenance of the chondrogenic phenotype, which, together with modulated physical properties, makes GH-GlcN gelatin-HA cryogel a suitable scaffold for cartilage tissue engineering In Vivo [43].

Biodegradable scaffolds were produced by free-radical polymerization of dextran modified with oligo L-lactide bearing HEMA end groups (HEMA-LLA-D cryogel) using MBA as a cross-linker [137]. In Vivo biodegradability and biocompatibility properties showed the potential of the HEMA-LLA-D cryogel-based scaffolds for the tissue engineering. Chondrocytes cultured in the cryogels rapidly proliferated and fully covered cryogel surfaces after nine days, along with significant secretion of ECM components at day 15, including collagen fibrils forming a mesh-like structure on the top of the scaffold [138].

A more complex approach was used in the work of Raina et al., where biocomposite cryogels consisting of silk, chitosan, agarose and HA were produced with and without bioactive glass [110]. The components were selected based on their properties, which will be added to the final biocomposite. Chitosan is biocompatible/hemocompatible with antibacterial effect, whereas silk is mechanically strong. Both HA and bioglass are known to have pro-osteogenic properties. Agarose was chosen because it can significantly improve the elastic properties of the matrix due to additional cross-links and the formation of interpenetrating networks, as well as enhance the proliferation of chondrocytes. It was also expected that chitosan, silk fibroin and HA would bind recombinant human bone morphogenic protein-2 (rhBMP-2), while HA would retain zoledronic acid, so this scaffold will be an optimal biodegradable carrier system for co-delivery of these bone active agents In Vivo. Animal studies demonstrate that the scaffold can be used as a replacement for bone grafts [110].

PVA-based cryogels are extremely well suited mechanically for vascular and cartilage tissue engineering applications. PVA/gelatin cryogels were made by freeze-thawing the mixture of the PVA and gelatin [139]. The gelatin was added to promote endothelial cell’s adhesion and proliferation. The cryogels did not have large pores, but that was not required for the application. On the contrary, a low porosity is beneficial in the sense that it ensures that the endothelial cells stay on the surface of the gel, rather than penetrating inside. The study showed that the application of a ramped shear stress on PVA/gelatin cryogels dramatically increases endothelial cell proliferation and facilitates neo-endothelialization, making cryogels particularly suitable for developing artificial arterial grafts [139]. Neo et al. combined PVA and silk in order to simultaneously improve the cell-hosting capability of PVA and the physical properties of silk cryogels for the replacement of nucleus pulposus in intervertebral discs [140].

DNA-based hydrogel scaffolds with an interpenetrating polymeric network were obtained by covalent cross-linking of DNA strands with a bifunctional cross-linker, polyethylene glycol diepoxide, at sub-zero temperatures. An additional alginate network was developed by adsorbing an alginate solution by the cryogel followed by ionic cross-linking with Ca^2+^. This significantly increased the cryogel toughness and energy dissipation compared to a single covalent network [141].

Composite materials are made not by the simple blending (mixing) of two or more polymers but by incorporating another type of material, such as nano-, micro- or macroparticles [76,142,143]. The final material will have unique characteristics that can be beneficial for biomedical applications [69,144,145].

Agarose cryogel loaded with silver nanoparticles coated with chitosan was fabricated using GA cross-linker. The obtained material demonstrated antibacterial activity against both Gram-positive and Gram-negative bacteria, as well as good compatibility with mammalian cells, characterized by sustained growth in a modified matrix, which makes this material useful for the engineering of soft tissues such as the pancreas, kidney, heart, and liver [144].

**Figure 5:**
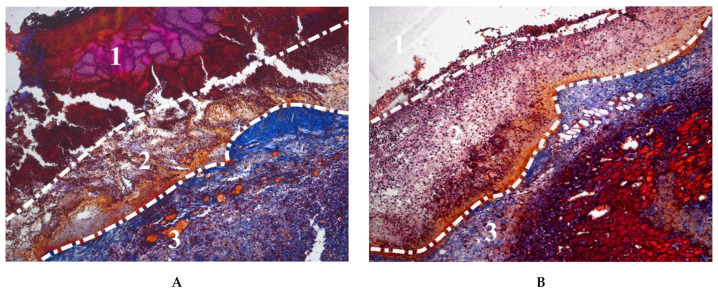
Microphotographs of Mallory-stained tissue sections from wounds treated with: (**A**) non-doped and (**B**) Zn-doped gelatin cryogel, at day 3 after trauma. (1) debris layer containing the cryogel surrounded by fibrous capsule, (2) initial granulation tissue, (3) mature granulation tissue.

In addition to being incorporated into cryogels in the form of nanoparticles, transition metals, as important microelements with various vital functions in living organisms, were entrapped into gelatin-based materials during cryotropic gelation. The incorporation of metals has been enhanced by adding pectin to the cryogel composition, which has a high affinity for divalent metals. The divalent ions of Zn, Cu, Mn and Co were effectively retained in the gelatin network. Cryogel with Zn^2+^ had significantly lower toxicity in vitro against human skin fibroblasts compared to soluble ions. In a rat excisional wound model, the Zn-doped cryogel exhibited faster resorption in contact with the wound bed compared to non-doped cryogels (Figure 5), which was attributed to increased biodegradation by Zn-dependent metalloproteinases, which play an important role in all phases of wound healing. Wound treatment with the Zn-doped cryogel resulted in accelerated skin regeneration accompanied by decreased leucocyte infiltration, rapid passing of inflammatory/proliferation phase and increased generation of dermis components [107]. 

PLLA cryogels have been reinforced by adding a bacterial cellulose fiber [68]. It had four times higher compressive strength and was more hydrophilic than original PLLA. A high strength hybrid scaffold was produced by combining a mechanically stable poly(lactic-co-glycolic acid) (PLGA) microspheres with a bioactive gelatin cryogel [146]. Both PLGA and gelatin were incorporated with a high percentage of nanohyroxyapatite (nHAP), in order to induce osteo-inductive and osteo-conductive properties. The hybrid scaffold showed 25-fold higher ultimate stress and 21-fold higher Young’s modulus than the cryogel scaffold, which makes it a good candidate for bone regeneration. In vitro studies using rabbit bone marrow-derived stem cells (rBMSCs) in the cryogel and hybrid scaffolds based on DNA content, alkaline phosphatase activity, and mineral deposition have shown a good cell proliferation and osteogenic differentiation of rBMSCs close to a natural environment.

In an attempt to facilitate supra-alveolar ridge augmentation, BMP-2-loaded PLGA microspheres were mechanically entrapped into gelatin/HA/β-tricalcium phosphate cryogel composite formed using GA cross-linker. Compared to the BMP-2-infused solution, the sustained release of BMP-2 from the microsphere-embedded matrix within four weeks of implantation was significantly more effective and promoted implant osteointegration by facilitating new bone deposition on the residual material [147].

Injectable antibacterial conductive cryogels based on carbon nanotube (CNT) and chitosan functionalized with glycidyl methacrylate have been synthesized [148]. The incorporation of CNT in the chitosan matrix resulted in a material with robust mechanical property, rapid shape recovery, fast blood absorption rate, and excellent sensitivity to near-infrared (NIR) stimuli. The great potential of these materials for In Vivo lethal non-compressible hemorrhage hemostasis and wound healing applications has been demonstrated [148]. In particular, the CNT-chitosan conductive dressing showed a higher wound healing efficiency than the non-CNT dressing. This was attributed to the transmission of electrical signals from the conductive cryogel dressing to the wound site, the activation of cellular activity, and the positive effect of CNT on the level of growth factors involved in the wound healing process. 

A two-layer cryogel wound dressing material was formed using polyvinylpyrrolidone (PVP) and gelatin as the external top and bottom layers, respectively. The incorporation of iodine in PVP layer allowed for its sustained release and gave the material antimicrobial property. Furthermore, the introduction of microparticles of gelatin loaded with mannose-6-phosphate and human fibrinogen into the gelatin layer improved the wound healing process by preventing scar formation and significantly accelerating skin regeneration and epidermis formation at two weeks after implantation in a rabbit model [149]. Similarly, a bilayer skin construct consisting of two layers of gelatin cryogels, embedded with sliver nanoparticles at the top layer and loaded with platelet-derived growth factor-BB (PDGF-BB) in the bottom layer, was developed. The release of silver nanoparticles gave the cryogel antibacterial activity against *Pseudomonas aeruginosa*, *Staphylococcus aureus* and *Escherichia coli*, while PDGF-BB accelerated re-epithelialization, the formation of granulation tissue and angiogenesis. Co-loaded scaffolds had a great potential for promoting diabetic wound healing in transgenic type II diabetic mice [150].

### 3.4. Stimuli Responsive Cryogels

Biomaterials that respond to external stimuli are of particular interest for the development of “smart” drug delivery systems, sensors and electro-conductive materials for regenerative medicine and tissue engineering. The stimuli-responsive biomaterials undergo significant changes in volume or other physical properties in response to stimulation by temperature, pH, electric, magnetic, or other environmental signals. 

The synthesis of electro-conductive cryogels from nanofillers 2-hydroxyethylcellulose and polyaniline by a combination of photochemical cross-linking and cryogenic treatment caused an increase in the electrical conductivity of the material as compared to the material without polyaniline nanoparticles. Composite cryogels have demonstrated high potential for use as scaffolds for tissue engineering by maintaining the viability and proliferation of L929 fibroblasts in addition to electrically controlled cell behavior, including adhesion, proliferation, morphological changes, and alignment in parallel with an electric field [78].

Biodegradable and thermos-sensitive HEMA/lactate/dextran cryogels were synthesized by adding *N*-isopropylacrylamide (NIPA) to the composition [151]. Varying the composition of NIPA and lactate dextran made it possible to control the swelling of cryogels, their degradation and drug release characteristics. The release profile of simvastatin loaded into HEMA/lactate/dextran cryogels depended on their composition and the temperature of the medium. NIPA/HEMA/lactate/dextran cryogels prepared using different loading methods of simvastatin can be used for controlled drug delivery to bone defects [151].

It has been shown that the development of a macroporous collagen cryogel using amino-functionalized graphene imparts electrically conductive properties to the material. These cryogels supported bone marrow mesenchymal stem cells (BM-MSC) growth and proliferation and their stemness upon electrical stimulation and further improved cell migration and proliferation during In Vivo transplantation into the injured spinal cord [90].

A glucose biosensor was made on the basis of a chitosan-bovine serum albumin cryogel with incorporated multiwalled carbon nanotubes (MWCNTs), ferrocene, and glucose oxidase [152]. The incorporation of glucose oxidase into the composite cryogel did not inhibit the enzymatic activity. The large surface area of the porous cryogel, combined with the good electron transfer property of MWCNTs, enables sensitive electrochemical detection of glucose. The electron transfer was mediated by ferrocene trapped in the cryogel and this helped to avoid any interfering responses. The biosensor demonstrated high operational stability after more than 350 injections (RSD = 3.6%), with a wide linear range from 0.010 to 30 mM and a low Michaelis-Menten constant (1.5 mM). The biosensor response to glucose was unaffected by dissolved oxygen and showed no response to common interferences in blood samples such as ascorbic acid and uric acid at physiological levels [152].

Tannic acid/ferric ions (TA/Fe^3+^) have been added to a chitosan/silk fibroin scaffold as a stimuli-responsive agent for photothermal therapy [153]. The photothermal agent TA/Fe^3+^ increased the surrounding temperature of the cryogel under NIR illumination and provided antimicrobial activity against both Gram-negative and Gram-positive bacteria, such as *Staphylococcus aureus* and *Escherichia coli*. Animal experiments have shown that this antibiotic-free cryogel is effective in killing microbes in a wound and accelerating wound healing, and is therefore a promising wound dressing material for clinical use. Photo-thermally active cryogels have been prepared for the release of antimicrobial peptides [154]. The peptides were covalently conjugated onto furan-based cryogel scaffolds by Diels-Alder cycloaddition and were enabled to release “on-demand” at NIR exposure to kill bacteria.

On the other hand, NIR-responsive PDA nanoparticles were used to modify chitosan/silk fibroin cryogel designed as a multifunctional material to regulate wound microenvironment. The cryogels showed efficient wound healing in skin defects in a rat model. Due to the photothermally enhanced antibacterial properties, which were demonstrated against *Escherichia coli* and *Staphylococcus* *aureus*, the cryogel can effectively prevent bacteria-induced infections in the wound area. The presence of reactive catechol groups on PDA nanoparticles provides sites for the adsorption of proteins and polypeptides including growth factors offering a significant enhancement of cell proliferation, recruitment, and tissue remodeling functions in the wound bed. Beyond that, these catechol groups are endowed with the ability to scavenge radicals and thus eliminate reactive oxygen species (ROS) overproduced during inflammatory responses [155].

While stimuli-responsive materials have some advantages, such as the control of drug release in response to the stimuli or electrical conductivity, they also include the addition of additives to the cryogel composition, which is not always biodegradable. This must be taken into account when developing biomaterials, and the possible harmful effects of additives and their fate in the body must be evaluated.

### 3.5. Modification/Functionalization of Cryogels to Enhance Cell Response

Despite the established value of scaffolds based on polymeric cryogel for the engineering of soft tissue, their modification by the inclusion of various additives may be necessary to induce a specific cell response, improve degradability and encourage tissue regeneration. This can be achieved by introducing various proteins, growth factors, peptides, and nanoparticles using well-designed surface chemistry in the scaffold structure to control its biological, mechanical and physical properties. 

The immobilization of the HEP component inside the cryogels allowed their secondary functionalization via non-covalent electrostatic interactions with various biological factors providing a suitable microenvironment required for cell growth and the regeneration of tissues. Injectable star-PEG-HEP cryogel beads with established compatibility for PC-12 and MSC culture were modified with HEP-binding growth factors such as glial cell-derived neurotrophic growth factor (GDNF) and nerve growth factor (NGF) for neuroprotection applications [96]. Similarly, HEP was conjugated to gelatin-based cryogels, followed by coating with vascular endothelial growth factor (VEGF) bearing HEP binding domains. This induced the controlled release of VEGF from the cryogel for functional angiogenesis with a high therapeutic effect on migration and tube formation by human umbilical vein endothelial cells (HUVEC) in vitro. In addition, a promoted vascularization of an ischemic hind limb model in mice has been observed [32]. Growth factors were also directly added to cryogels during preparation. Basic fibroblast growth factor (bFGF) has been incorporated into chitosan-gluconic acid conjugate/PVA cryogels by physical entrapment to accelerate wound healing. The increase in the healing rate of the wounds covered with these cryogels was observed compared to cryogels without bFGF, suggesting these cryogels as a promising wound care material [156].

Cell adhesion is a key process for the success of scaffold-based tissue regeneration strategies. In tissues, cell adhesion is primarily mediated by interactions between cell receptors, particularly integrins, and adhesive proteins present in ECM-regulating cell motility, growth, morphology, and differentiation. The most common minimal binding site recognized by integrins is the tripeptide RGD, which is mainly found in fibronectin and vitronectin [157]. It has been found that the introduction of short peptide sequences containing RGD motif into cryogel materials using various immobilization methods was sufficient to maintain cell adhesion, proliferation and regulation of various cellular functions. Macroporous alginate cryogels with introduced pendant methacrylate groups were covalently modified with cell adhesive RGD ligands via covalent coupling to polymerizable acryloyl-PEG-NHS. The presence of RGD enhanced the attachment and proliferation of MSCs compared to unmodified cryogels, which was important in preventing anoikis and cell retention during syringe injection of cell-seeded cryogels and, therefore, for increasing survival and promoting cell engraftment In Vivo [158]. Covalent functionalization of starPEG-HEP cryogel with cRGDyK cyclic peptide sequence was achieved by carbodiimide chemistry involving carboxyl groups of heparin, which were pre-activated with EDC/sulfo-NHS before exposing to peptides. The peptide-activated material was shown to support endothelial cell attachment, migration and proliferation [95]. Similarly, another study demonstrated that the functionalization of starPEG-HEP cryogels with RGD, using the latter strategy, resulted in the formation of scaffolds that support MSC culture. MSCs seeded in the peptide-modified material retained their ability to secrete ECM proteins, providing biological cues for further pancreatic islets cultivation. Islets inside of cryogel have demonstrated continued survival, function and proliferation in vitro and In Vivo in a mouse model [134]. 

PEG-heparin cryogels were used as a cell-housing scaffold for transplantation of gene-modified MSCs capable of sustained secretion of anti-CD33-anti-CD3 bispecific antibody. A macroporous material functionalized with an RGD-containing peptide sequence (H_2_N-GWGGRGDSP-CONH_2_) has been shown to support the survival and proliferation of the stromal cells upon subcutaneous implantation of cryogels in tumor-bearing mice. The implanted cells were able to constantly release high levels of antibodies sufficient to trigger T-cell-mediated anti-tumor response and regression of blasts of acute myeloid leukemia [97].

In another attempt to improve cell attachment on hybrid cryogel composed of HA, alginate and gelatin, RGD in the form of acrylated peptide sequence (ACRL-PEG-G_4_RGDSP) was incorporated into the material using radical polymerization technique. The covalent bond formed between the acryl groups in the peptides and methacrylated polymers in the cryogel. 3T3 fibroblasts cultured in functionalized cryogels were characterized by improved cell adherence, homogenous distribution, and spindle-shaped morphology [159]. The application of a similar immobilization technique for the functionalization of synthetic PEG-based cryogels with RGDS peptide resulted in a comparable ability of the scaffold to support cell attachment, infiltration and viability, especially at higher ligand concentrations [67]. Comparable cell behavior was detected in chitosan cryogels functionalized with an RGD-containing peptide (GRGDGY), where the human fibroblasts attachment as well as HUVEC spreading increased in proportion to the surface concentration of RGD [160]. 

Besides RGD, other short peptide ligands such as laminin-derived YIGSR and IKVAV or fibronectin-derived REDV and LDV, among others, are also known to modulate cellular responses in biomimetic hydrogel materials. Synthetic oligopeptides are capable of reproducing minimal bioactive peptide sequences of full-length growth factors and ECM components, and are therefore a promising alternative to natural proteins in tissue regeneration. In contrast to their parent molecules, which can undergo rapid denaturation, synthetic peptides are more stable and soluble, and their small size allows them to be effectively incorporated into scaffold materials at high concentrations. It is noteworthy that different cellular responses can be induced in a controllable manner according to the identity of introduced sequence. Peptides containing RGD and IKVAV motifs—namely, GRGDS-PASSKG_4_SRL_6_KK(maleimide)G and (maleimide)-GRKQAAS-IKVAV-SG4SRL_6_-KKG—were grafted to furan-modified HA/PEG cryogels via Diels-Alder reaction between furans and maleimide of the peptides. The cellular response was successfully controlled by immobilizing the biomimetic ligands. When luminal epithelial breast cancer T47D cells were cultured on RGD-modified cryogels, they spread out and adhered to the cryogels. Substituting RGD with IKVAV triggered the formation of 3D cell spheroid structures, similar to that observed In Vivo [161].

Although the combination of different ligands on the surface of hydrogel materials may be promising in enhancing their regenerative properties [162], this strategy has been poorly investigated. Recently, pHEMA-PEG cryogels with copolymerized β-cyclodextrin units were prepared as inert macroporous scaffolds, which were non-covalently modified with adamantylated Ada-Ahx-GGRGD and Ada-Ahx-GGGHK peptides via host-guest interactions. This affinity immobilization of the ligands made it possible to avoid unpredicted side chemical modifications of the material that can be observed during covalent attachment. This study demonstrates the feasibility of establishing specific cellular responses to the immobilized peptide signals in an inert 3D environment. The synergistic activity of RGD and GHK motifs was revealed in relation to the cell proliferation and behavior of 3T3 and PC-12 cells seeded on cryogel [163]. Furthermore, co-modification of cryogels with RGD and GHK, and then with Cu^2+^, enhanced regenerative angiogenic responses in vitro by a sharp increase in the cell proliferation, differentiation, and production of a number of cytokines related to angiogenesis and growth factors in HUVECs [164]. Although the proposed synthetic ECM-mimicking cryogel scaffold is non-biodegradable, it has proved to be an informative tool for studying multiple cell-responsive factors. Its use for tissue engineering will require the introduction of biodegradable units into the cryogel structure, which could also be of peptide nature. For this purpose, protease-sensitive peptides such as GGGPQGIWGQGK may be co-polymerized into the cryogel backbone to render them responsive to cellular proteases such as secreted metalloproteinases MMP-2 and MMP-9 [162].

Embedding multifunctional particles in the material network is another cryogel activation strategy. This can add additional biological/physical properties to the cryogel inherited from these components, which can improve the binding capacity of some active molecules, increase the specific surface area, modulate mechanical strength and induce different cellular responses. Coating of collagen cryogels with PDA nanoparticles facilitated adhesion of adipose tissue-derived MSCs cell, increasing their viability and proliferation compared to uncoated materials. The effect of cryogel modification is explained by providing a favorable environment for cell spreading and adhesion by PDA nanostructures owing to their ability to bind ECM components. Similarly, PDA nanoparticles can also be exploited for further functionalization of cryogels with ECM molecules [103]. Furthermore, the functional groups of mussel-inspired dopamine can act as a cross-linker to incorporate polypyrrole nanoparticles, gelatin-methacrylate, and PEGDA into a hybrid cryogel. The incorporated nanoparticles significantly improved the behavior of cardiomyocytes in the cryogels by increasing the expression of α-actinin and connexin-43, both of which are essential for the maturation of the myocardium and synchronous contraction. After implantation of the cryogel with cardiomyocytes onto infarcted myocardium in the hearts of rats, it was proven to reduce the infarct size and improve the cardiac function [165].

To trigger the osteogenic ability of a cryogel scaffold, various additives/bioactive factors could be added to the composition, among which hydroxyapatite nanoparticles are the most widely used inorganic biocompatible material. Type I collagen cryogel scaffold with entrapped nanocrystalline hydroxyapatite was able to support human bone marrow stromal cells (HBMSCs) survival, proliferation, differentiation and individual functionality in a monoculture system for 21 days. The composite cryogel promoted tissue regeneration with no adverse reactions; thus, mature bone formation including lacunae and mineralization was prominent after 12 weeks of subcutaneous and bone implantations in rats [91]. Nanoparticles of Zn- and Ce-substituted hydroxyapatite were incorporated into poly(hydroxypropyl methacrylate) biodegradable cryogels owing to their bioactivity and antibacterial properties. In vitro tests confirmed that osteoblast cells (MG-63) show strong adhesion and growth in an attempt to create a ceaseless cell layer in the nano-composite cryogels while the measures of live microbes were clearly lower. Evidently, endochondral ossification detected In Vivo makes this material promising for clinical use in bone tissue engineering [166]. As another application, hydroxyapatite nanoparticles added to cryogel scaffold produced from silk-fibroin, chitosan, and agarose have been used for the controlled release of growth factors [110]. The cryogel was modified with recombinant human bone morphogenic protein-2 (rhBMP-2) in combination with zoledronic acid to enhance osteoinduction and inhibit bone resorption, respectively. Both factors were introduced to the material via surface adsorption owing to their affinity to the hydroxyapatite component. Activated cryogels exhibited sustained proliferation of both C2C12 cells and MSCs for a period of six weeks with enhanced regulation of their phenotype towards osteogenic differentiation. An In Vivo study has clearly demonstrated the ability of the developed material to accelerate bone regeneration [110].

Other studies have demonstrated the useful application of particle-embedded cryogels, beyond hydroxyapatite, for the effective release and delivery of therapeutics. Dwivedi et al. conducted a study where chitosan-gelatin cryogels were modified by the incorporation of gelatin microspheres loaded with chondroitin sulfate. These microparticles were embedded in the cryogels so that they can deliver the drug with preserved activity to goat primary chondrocytes in an appropriate manner with controlled release and longer duration of action. As chondroitin sulfate encourages chondrocyte proliferation and differentiation, the materials developed hold great promise for cartilage tissue engineering [122]. In an attempt to promote wound healing, cerium oxide nanoparticles conjugated with miRNA146a were loaded into cryogels prepared from [2-(methacryloloxy)ethyl]dimethyl-(3-sulfopropyl) ammonium hydroxide) or 3-[[2-(methacryloyloxy)ethyl] dimethylammonio] propionate and 2-hydroxyethyl methacrylate. Owing to their radical scavenging properties, cerium oxide nanoparticles can eliminate oxidative stress in diabetic wounds in addition to their effective delivery of miRNA146a contributing to accelerated wound healing in diabetic mice [167].

## 4. Characterization of the Degradation Process In Vitro and In Vivo

The degradation and resorption of biomaterials is critical for both drug delivery and tissue engineering applications. The gradual replacement of the implanted scaffold with newly formed tissues underlies the normal healing process, which should avoid chronic inflammation, fibrotic encapsulation and implant failure [168]. The improved 3D structure of the macroporous cryogels is capable of absorbing a large amount of aqueous solution or body fluids and effectively supports cell infiltration, which should promote both enzymatic and non-enzymatic degradation [169]. They undergo degradation through hydrolysis or other mechanisms that are regulated by many factors: the nature of polymer, the type and degree of cross-linking, porosity, and others. The pH of degradation media is an important parameter to be concerned about during cryogel development, as its shifting to lower values can cause local tissue necrosis due to pH-induced autocatalytic degradation [81,170]. 

In vitro degradation is usually studied in a buffer or simulated biological media by analyzing the mass loss of the scaffold over time [48,85,151]. The simulated degradation conditions may involve chemical hydrolysis, such as in the case of PLLA scaffold [151,170], or induced by adding enzymes [69]. Temperature, pH and cryogel composition play a key role in the degradation profile. For example, the degradation rate of the HEMA-lactate-dextran-co-NIPA scaffold increased with temperature due to the hydrolysis of lactate spacer and was higher for scaffolds with a large proportion of HEMA-lactate-dextran component [151]. The degradation of inulin cryogels was dependent on the concentration of cross-linker, DVS, and pH [85]. The degradation of inulin cryogels was examined in different buffer solutions to predict drug release properties of the materials under different biological conditions, such as pH 1, pH 7.4 and pH 9 and a temperature of 37.5 °C. The full degradation of inulin cryogels was observed at pH 1, and it took between one and six days depending on the degree of cross-linking. The degradation at a higher pH required a longer time and was lower for the cryogels with a higher degree of cross-linking. Thus, at the lowest cross-linking degree (75 mol%) after 21 days, the inulin cryogel had 61% and 45% weight losses at pH 7.4 and pH 9, respectively.

The degradation of chitosan cryogels prepared using oxidized dextran cross-linking was shown to be faster in PBS (pH 7.4) in the presence of higher dextran content due to the hydrophilicity and lower molecular weight of dextran molecules compared to chitosan. The reduction of Schiff’s bases in the cross-linked cryogels by sodium borohydride resulted in a lower degradation degree compared to the non-treated ones since the latter have pH-sensitive hemiacetal and hemiaminal groups [51]. Schiff’s bases formation was exploited for the cross-linking of chitosan cryogels using biodegradable polyurethane nanoparticles as cross-linkers. The nanoparticles modification with poly(1,4-butylene adipate) diol resulted in an increased susceptibility to the degradation of cryogels with 22.9% weight loss after 28 days in PBS. The obtained cryogels were pH-sensitive and could be liquefied when exposed to acetic acid (pH < 1) or in the presence of aniline with monoamine group (pH ~10) [171]. These examples show that degradation is a complex process and both environmental conditions and composition of cryogels need to be considered. 

In the body, the polymer scaffolds degrade upon contact with enzymes; therefore, enzyme solutions are used to simulate the biological environment in which the scaffold will be located. HA microneedles-laden collagen cryogels cross-linked via 4s-arm-PEG succinimidyl glutarate and loaded with moxifloxacin were studied using collagenase-containing media and simulated tear fluid media (STF) composed of lysozyme, d-glucose, sodium chloride, bovine serum albumin and CaCl_2_·2H_2_O. The cryogels were completely degraded within 24 h in case of collagenase while the degradation lasted about one month in STF [172]. The degradation of collagen cryogels fabricated using amine-functionalized graphene-mediated linking or EDC/NHS linking was estimated in collagenase type I solution. It was shown that the graphene-cross-linked scaffolds were characterized by increased proteolytic resistance [90].

Solutions of several enzymes were used to identify degradation depending on the type of enzyme and its concentration. The degradation of gelatin/HA/glucosamine cryogels was studied using hyaluronidase and collagenase mixed solutions [43]. It increased with increasing glucosamine content. Collagenase induced a more effective dissolution of materials, which were completely degraded within 4 h, while in hyaluronidase solution, the degradation degree was only 35% after two days. The higher degradation in the presence of glucosamine could be a result of the reduced cross-linking density with increased content of glucosamine. A small molecule of glucosamine consists of glucose with one amine group, which cannot facilitate cross-linking with HA in the same way as gelatin, which contains multiple primary amino groups. The degradation was more pronounced in collagenase solution since gelatin was the major component in the cryogel with >80 wt% [43]. Analysis of degradation of gelatin and gelatin-HA in collagenase solution showed faster degradation of gelatin-HA compared to gelatin cryogels. Since collagenase only hydrolyzes peptide bonds in gelatin, replacing gelatin with HA leads to a more rapid degradation of gelatin-HA cryogels [106]. The degradation of collagen/HA/gelatin scaffolds was dependent on the enzyme used and its concentration [44]. Lysozyme decomposed the collagen/HA/gelatin scaffold gradually in one week and about 38% and 36% of the original weight remained after seven days of treatment with 10,000 and 30,000 U/mL of lysozyme, respectively. Scaffold degrades completely in 50 U/mL hyaluronidase solution after seven days and in 20 U/mL collagenase solution after 3 h. 

It was observed that an inner morphological structure of hydrogels influences their swelling and degradation behavior. In vitro degradation behavior of the gelatin-chitosan hydrogels prepared by freeze-drying was investigated for up to five weeks using collagenase, lysozyme, and N-acetyl-β-D-glucosaminidase [81]. The weight-losses of hydrogels and porous 3D hydrogels, their degradation products identified by UV-Vis spectroscopy and high-performance liquid chromatography (HPLC) and changes in the pH were analyzed. Porous 3D hydrogels with bigger pores provide a larger surface area and more active sites for enzymes to react upon, and thus more gelatin molecules in the hydrogels can be cleaved and a higher weigh loss have been observed. The degradation was additionally reduced by in-gel-embedded α-tocopherol because of hydrophobic interactions with their constituents, and hindering the effect. Gelatin/dopamine cryogel degradation was dependent on the dopamine content. Increased dopamine concentration led to the formation of a greater number of cross-links in the cryogel network, which increased its biodegradation period [173]. Likewise, the use of oxidized dextran at a concentration of 4% rather than 0.5% in PL cryogels increased the time of their complete degradation from 90 to 240 days [108]. 

Papain treatments are often used to mimic enzymatic activity occurring In Vivo during wound healing. The influence of papain on the degradation of poly((N-5-(2-hydroxyethyl)-L-glutamine)/HEMA cryogels was tested via rheological analysis. The fast degradation of the material was revealed by a decrease of its storage modulus up to 50% during in first day of incubation [174]. The degradation of gelatin/ascorbic acid cryogels was assessed in the presence of MMP-1 for 28 days at 37 °C. A significant decrease in the weight of the samples was found, confirming the biodegradability of the carrier materials, which allows the delivery of ascorbic acid [175]. In other studies, the percentage of degradation of the PVA-starch/Ag-HAP cryogel increased significantly with the activity of the α-amylase enzyme in solution (>600 IU/mL) [69].

In Vivo degradation is a much more complex process that depends on the environment and cell activity. A tissue-specific local degradation of collagen/HAP was reported. Upon subcutaneous implantation, the scaffold degradation was observed after four weeks, whereas it required 8 to 12 weeks in the case of bone implantation [91]. Gelatin/HAP/calcium sulphate cryogels containing BMP-2 and zoledronic acid (ZA) were studied using an abdominal muscle pouch model. The histological analysis showed that although ZA-modified scaffolds had a slower degradation rate compared to those modified with BMP-2 solely, they appeared to be better infiltrated with bone tissues [176]. 

Degradation of implanted HEMA-LLA-dextran cryogels occurred faster in soft (subcutaneous, iliac submuscular, auricular) tissues compared to bone (calvarial) tissues [137]. The degradation started from the periphery and preceded to the central area of the implanted cryogels, allowing newly formed collagen fibers to be deposited within the scaffold. Residual particulate products of cryogel degradation were still detected six months after implantation. In Vivo resorption and a change in morphology of four different cryogels made of poly(vinyl caprolactam) (PVC), PVA-alginate-bioactive glass (PVA-Alg-glass) composite, pHEMA-gelatin and chitosan-agarose-gelatin were studied after six weeks of subcutaneous implantation in C57Bl/10.Q inbred mice by scanning electron microscopy [119]. PVA-Alg-glass cryogel was found to degrade faster compared to other materials. PVA-Alg-glass and PVC cryogels were degraded, respectively, by ca. 85 and 75% of their initial amount, whereas the degradation of pHEMA-gelatin and chitosan-agarose-gelatin cryogels was delayed with only a 40% resorption rate for both the materials [119]. 

However, the degradation products of biocompatible materials, which are generally considered biologically inert, can have local stimulatory or adverse effects In Vivo. Hydrolysis of cryogels based on aliphatic polyesters such as PLGA is accompanied by the generation of acidic products decreasing cell mobility angiogenesis due to local decrease in the pH. It was found that a fast degradation negatively affected cell viability and migration into the scaffold in vitro and In Vivo due to significant acidification of the local environment as a result of polymer degradation [170]. 

## 5. Application of Biodegradable Cryogels

Cryogels, due to the macroporous structure of interconnected pores and shape memory together with customizable physic-mechanical properties, have generated great interest in the biomedical community for tissue engineering, as carriers for the delivery of drugs, vaccines, or even whole cells. Several comprehensive reviews on the application of cryogels have been recently published [4,5,7,177] and have been discussed in the previous sections. Table 3 provides a summary of the areas, where cryogels have been used, with examples of applications for biodegradable cryogels. 

## 6. Concluding Remarks

Recent studies have demonstrated that cryogels are a versatile platform for the design of various biomaterials to deal with emerging biomedical problems. The overview of the literature shows that interest in the use of biodegradable materials is increasing. Most biodegradable polymers are biocompatible, eco-friendly, and renewable. Some studies do not pay much attention to the study of biodegradation and suggest that a material is biodegradable depending on the material from which it is made. On the other hand, studies evaluating biodegradability show that it plays an important role in tissue engineering design. We believe that biodegradability is a key property of materials used to provide better solutions to the design of 3D tissue constructs and drug delivery systems. Better understanding of the biodegradation process will allow us to design advanced materials that control the release of drugs into a particular part of the body. Better control of the degradation of 3D tissue constructs will also facilitate the healing process and replacement with healthy tissue. 

Cryogels have been proven to provide a versatile platform for the development of biomedical devices due to their unique three-dimensional macroporous structure, adjustable physical and mechanical properties, and their ability to mimic natural tissues. A distinct advantage is the ability to provide additional functionalities through chemical modification or the inclusion of particles/polymers in the cryogel composition that add new biological function and promote regeneration. Future work should focus on creating materials with biochemical and biophysical cues to the cell, to improve their adhesion, proliferation, and differentiation. As has already been demonstrated, modification with certain bioactive molecules significantly improves the performance of the scaffold. The incorporation of specific cell recognition/signaling molecules into cryogel scaffolds is a promising and challenging task that essentially depends on the propensity of biomaterial for modification and the effectiveness of the applied immobilization technique. Depending on the desired application, most of the currently used functionalization strategies involve the addition of growth factors, ECM proteins, large polypeptides, and small peptide motifs. In particular, cryogels activated by conjugation of defined protein fragments or short peptides to a polymer backbone can be designed to mimic some essential/specific features of native ECM, with both stable and predictable outcomes. The impact of physical properties (pore size, interconnectivity, mechanical properties) on both cell behavior and the degradation process also requires further study. 

## Figures and Tables

**Table 1:** Types of the cross-linking reactions for the formation of cryogel.

Cross-Linking Type	Methods	Examples	References
Physical cross-linking	Ionic interaction	Alginate cryogel cross-linked with Ca^2+^	[19]
		Ionic cryotropic gelation of pectin-chitosan	[20]
	Freezing-thawing cycle	PVA-gelatin cryogel; hydrogen bonding, formation of crystallites of PVA	[21]
		Agarose cryogel, intramolecular and intermolecular hydrogen bonding	[22]
		Chitosan cryogel, formation of crystallites composed of D-glucosamine and N-acetylglucosamine units	[23,24]
Chemical cross-linking	Reaction between the functional groups of polymer and cross-linker or polymer–polymer	Condensation of an amine with the carbonyl group, formation of Schiff bases; chitosan and gelatin cross-linked with glutaraldehyde.	[25,26,27,28,29]
		HEMA-Gelatin: radical polymerization and cross-linking with poly(ethylene glycol) diacrylate (PEGDA)	[30]
		Reactions of multifunctional-amine and epoxy groups; chitosan and diglycidyl ethers of ethylene glycol (EGDGE) or polyethylene glycol (PEGDGE)	[31]
		Reaction between amine and carboxy groups: gelatin, gelatin-heparin cross-linked with EDC/NHS	[32,33]
		Oxime-cross-linking of gelatin-oxyamine and hyaluronan-aldehyde; gelatin-hyaluronan cryogel	[34]
	Radiation cross- linking, exposure to high energy source (electron beam)	Electron-beam initiated cross-linking reaction; dextran, hyaluronan	[35,36,37]
	Photo-cross-linking	UV photo-cross-linking; hyaluronic acid, dextran	[38,39]

**Table 2:** Natural polymers used for cryogel preparation.

	Biopolymers	Descriptions	Functionalities	Examples of Cross-Linking/ References
Polysaccharides	Amylopectin, amylose and starch	Neutrally charged, composed of glucose units	hydroxyl	Freezing-thawing [69,70]
	Alginate	Anionic polyelectrolyte, composed of D-mannuronate and L-guluronate	carboxylate and hydroxyl	Cross-linking with Ca^2+^ [19]; redox radical polymerization of methacrylated alginate [71,72,73]; adipic acid dihydrazide/EDC [74]; Alginate beads [75]
	Carrageenan	Anionic polyelectrolyte, composed of D-galactose and 3,6-anhydro-D-galactose	hydroxyl and sulfate	GA, EDC/NHS, ginipin [45,46]; glyoxal [76]
	Cellulose	Neutrally charged, composed of D-glucose	hydroxyl	UV radiation using H_2_O_2_ as a photo-initiator [55]. Cellulose particles as filler [68,76,77,78]
	Chitosan	Cationic polyelectrolyte, composed of D-glucosamine and N-acetyl-D-glucosamine	amine and hydroxyl	GA [26,41,79,80]; EDS/NHS [81]; dextran modified with dialdehyde groups [51,52]; diglycidyl ethers [31]; ionic non-covalent cross-linking [40,41].
	Dextran	Neutrally charged, composed of D-glucopyranose	hydroxyl	Polymerization of acrylated dextran [35,36,37,82,83]; DVS [84]
	Inulin	Neutrally charged, composed of chain-terminating glucosyl moieties and a repetitive fructosyl moiety, which are linked by β(2,1) bonds.	hydroxyl	DVS [85]
	Gellan gum	Anionic polyelectrolyte, composed of D-glucose, D-glucuronic acid and L-rhamnose	carboxylate and hydroxyl	Physical cross-linking, freeze-dried [86] in composition with alginate gel, Ca^2+^, freeze-drying [87]
	Locust bean gum	Neutrally charged, composed of (1-4) linked beta-D mannose and (1-6) linked alpha-D galactose.	hydroxyl	Physical cross-linking, freeze–thawing [88,89]
	Methylcellulose	Neutrally charged, composed of D- glucose	hydroxyl	Adipic dihydrazide cross-linking chemistry [90,91]
	Pectin	Anionic polyelectrolyte, composed of homogalacturonan, rhamnogalacturonan-I and rhamnogalacturonan-II	carboxylate and hydroxyl	Physically cross-linked pectin [92]; ionic cryotropic gelation method of pectin and chitosan [20,93]
Glycosaminoglycans	Chondroitin sulfate	Anionic polyelectrolyte, composed of D-glucuronic acid and N-acetyl-D-galactosamine	amide, carboxylate, hydroxyl and sulfate	methacrylated chondroitin sulfate was cross-linked along with PEGDA via free radical polymerization [94]
	Heparin	Anionic polyelectrolyte, composed of D-glucuronic acid, D-glucosamine, L-iduronic acid and N-acetyl-D-glucosamine	amide, carboxylate, hydroxyl and sulfate	EDC/sulfo-NHS, PEG and heparin [95,96,97]
	Hyaluronic acid	Anionic polyelectrolyte, composed of D-glucuronic acid and N-acetyl-D-glucosamine	amide, carboxylate and hydroxyl	Methacrylated hyaluronic acid/PEGDA, free radical polymerization [94]; glycidyl methacrylate [98]; UV photo-cross-linking of HA methacrylate [39];
	Pullulan	Neutrally charged, composed of maltotriose	hydroxyl	Physical, composite with PVA [99]
Polypeptides/proteins	Albumin	Amphoteric polyelectrolyte, composed of various amino acids	amine, carboxylate and hydroxyl	Denaturants (urea) and thiol-bearing reductants (cysteine), formation of intermolecular disulfide bridges in the junction nodes [100,101]
	Casein	Amphoteric polyelectrolyte, composed of various amino acids	amine, carboxylate and phosphate	Physically cross-linked, composite with PVA, freezing–thawing [102]
	Collagen	Amphoteric polyelectrolyte, composed of various amino acids	amine, carboxylate and hydroxyl	EDC/NHS [103,104]; amino-functionalized graphene as a nano-cross-linking agent [90]
	Elastin	Amphoteric polyelectrolyte, composed of various amino acids	amine and carboxylate	Composite with CNT and iron oxide [105]
	Fibrinogen	Amphoteric polyelectrolyte, a glycoprotein complex	amine, carboxylate and hydroxyl	GA [28,50]
	Gelatin	Amphoteric polyelectrolyte, composed of various amino acids	amine, carboxylate and hydroxyl	EDC [106]; GA [27,28,50,107]
	Platelet lysate (blood)	Amphoteric polyelectrolyte, composed of various amino acids	amine, carboxylate and hydroxyl	platelet lysate (PL) and aldehyde-functionalized cellulose nanocrystals [77]; Pl and oxidized dextran [108]
	Silk fibroin	Amphoteric polyelectrolyte, composed of various amino acids	amine, carboxylate and hydroxyl	Silk fibroin physical cross-linking [8]; cross-linked with butanediol diglycidyl ether [109]; Composite with gelatin, chitosan, collagen [110,111]

**Table 3:** The main applications of biodegradable cryogels with examples.

Application	Material/Polymer	Description	Ref
Bone regeneration	Gelatin-hydroxyapatite	Controlled release of human bone morphogenic protein-2 from the scaffold supports long term proliferation and differentiation of MSCs and accelerates In Vivo bone regeneration	[110]
		VEGF-loaded gelatin scaffold yields increased repair of critical-sized bone defects in the proximal tibiae and early phase of fracture healing	[178]
	Poly(lactic-co-glycolic acid) (PLGA)/ nanohydroxyapatite (nHAP) microsphere avity fitted with gelatin/nHAP cryogel	Embedding of PLGA/nHAP microsphere stimulates proliferation and osteogenic differentiation of rBMSCs and regeneration of mid-diaphyseal tibia defects in rabbits	[146]
	HEMA-lactate-Dextran-co-NIPA	Controlled degradation of the thermoresponsive cryogel scaffold permits the release of simvastatin for regeneration of bone defects	[151]
	Gelatin cryogel inside poly-epsilon-caprolactone scaffold	Bone tissue engineering.	[179]
Cartilage	Chitosan-agarose-gelatin	Supports adhesion, growth and secretion of ECM by chondrocytes and demonstrates suitability for In Vivo implantation during the development of osteoarthritis	[180]
	HEMA-gelatin	Cultured chondrocytes grow in the cryogel over 3 weeks with increased metabolic activity and collagen type-II gene expression	[181]
	HEMA-lactate-dextran	Supports rapid proliferation of bovine articular chondrocytes and secretion of collagen fibrils	[138]
	PEG cross-linked with chondroitin sulfate or hyaluronic acid	Contributes to specific ECM proteins secretion in chondrocyte-seeded cryogels, stimulates glycosamino-glycan or collagen II accumulation.	[94]
	Chitosan-gelatin with incorporated chondroitin sulfate-loaded gelatin-microspheres	Controlled release of the drug enhancing chondrocyte proliferation and differentiation	[122]
Nerve regeneration	3D engineered nerve conduit consisting of gelatin cryogel	Assists peripheral nerve regeneration after neurorrhaphy and prevents the invasion of scar tissue in rat sciatic nerve transected model	[33]
	Aligned chitosan-gelatin cryogel filler in polyurethane nerve conduit	Provides interconnected porous channels for directional regeneration of axons and cues for Schwann cells adhesion and migration	[60]
	Injectable Laminin-coated cryogel of Alginate-carboxymethyl-cellulose	Supports neuronal cell adhesion and spreading, while its mechanical properties offer protection of cell viability and differentiated morphology as well as scaffold integrity during syringe injection	[182]
	Electroconductive Collagen based cryogel cross-linked with graphene	Supports BM-MSCs growth and proliferation of and their stemness upon electric stimulation	[90]
	Injectable PEG-heparin cryogel microcarriers functionalized with GDNF and NGF neuroprotective factors	Allows the proliferation and differentiation of the neuronal cells and conserves their viability upon injection for applications in neurodegenerative diseases	[96]
	Amino-functionalized graphene cross-linked collagen cryogel	Stem cell transplantation and neural tissue regeneration	[90]
	Dextran or gelatin-based cryogels linked to laminin	Promote the formation of artificial neural tissue in vitro by inducing human cord blood derived stem cell differentiation. Shows no inflammation or glial scarring when transplanted into a rat brain and promotes host neuroblasts infiltration.	[25]
Wound repair, skin regeneration	Gelatin	Supports formation of neoepidermal layer by the seeded keratinocytes in vitro and supports wound healing via cellular infiltration and biointegration in a porcine model	[27]
	3D printed gelatin scaffolds with silver nanoparticles and PDGF-BB	Shows potential for diabetic wound healing, re-epithelization, granulation tissue formation and angiogenesis in addition to antibacterial activity	[150]
	Gelatin cryogel with Zn metals	Promotes wound healing process and skin regeneration inducing intense dermis formation	[107]
	Polyvinylpyrrolidone-iodine-gelatin with microparticles loaded with mannose-6-phosphate and human fibrinogen	Prevents scar formation and accelerates skin regeneration while sustained release of iodine suppresses microbial growth	[149]
	Chitosan-silk fibroin with addition of tannic acid/ferric ion complex	Shows photothermal antimicrobial activity with high hemostatic and skin regenerative abilities	[153]
	Collagen-hyaluronic acid-gelatin	Co-culturing of keratinocytes, melanocytes and dermal fibroblasts results in normal human skin layer distribution. The scaffold ameliorates wound healing by decreasing neutrophil infiltration and thickening newly generated skin	[44]
	Gelatin	Bilayer wound dressing and skin regenerating graft for the treatment of surgically created full thickness wounds	[149]
	Chitosan and epsilon-poly lysine based cryogels	Multifunctional wound dressings for bleeding control and bacteria-infected wound healing.	[183]
	Polyurethane based cryogel with exosome.	Release of antioxidant, antibacterial wound dressing for diabetic and infectious wound healing.	[184]
Pancreatic/ Liver tissue engineering	PEG-heparin	MSC cultured on RGD-modified cryogel secrete ECM proteins providing cues for pancreatic islets and maintaining their survival and function In Vivo upon implantation	[134]
	PEG-alginate-gelatin	Supports proliferation and functionality of hepatic cells as spheroids cultured on 3D matrix	[185]
	Agarose-chitosan	Presents potential as in vitro liver tissue model supporting primary hepatocytes proliferation with increased cellular metabolic activity, albumin secretion and expression of hepatic CYP450 biomarker	[118]
Cardiac	HEMA-gelatin	Provides native environment for myocardial cells proliferation and differentiation with increased metabolic activity and formation of tubular structure	[186]
	Gelatin-PEGDA with polypyrrole nanoparticles	Induces the expression of α-actinin and connexin-43 in cardiomyocytes seeded in cryogels and improves the cardiac function after implantation onto infarcted myocardium	[165]
Vaccine delivery	Alginate	Transplanting of antigen-bearing tumor cells along with macrophage colony-stimulating factor and cytosine-phosphodiester-guanine oligodeoxynucleotide led to strong antigen-specific cellular and humoral responses In Vivo	[71,72,187]
Stem cell therapy	Collagen based cryogel bioscaffold coated with nanostructured polydopamine	A platform for mesenchymal stem cell therapy	[96,103]
Cell delivery	Injectable PEG based microcryogels with alginate.	For mesenchymal stromal cells delivery and alleviation of canine disc degeneration	[135,136]
Cancer research	Gelatin	Facilitates cancer cell invasion, migration and proliferation allowing for comprehensive characteristic of tumor cell behavior and testing the activity of anticancer drugs	[29]
	Hyaluronic acid-PEG	Immobilized biomimetic ligands offer control over cellular response resulting in enhanced spreading with RGD and formation of 3D cell spheroids with IKVAV	[161]
	HEMA-Alginate-Gelatin	3D culturing of prostate cancer cells and spheroids to study their progression and behavior	[188]
	Poly(ethylene glycol) diacrylate and type I collagen semi-interpenetrating polymer network cryogels	Tunable off-the-shelf platforms for 3D cancer cell	[112]
Drug delivery	HEMA-Gelatin	pH-responsive release of doxorubicin with increased rate in more acidic conditions mimicking tumor environment	[30]
	Inulin cryogel	Release of amoxicillin trihydrate	[85]
	Alginate beads	Release of quercetin as antioxidant	[19]
	Chitosan, hydroxyapatite, heparin, and polyvinyl alcohol composite	Release of bone morphogenic protein 2 and support of the differentiation of rat bone marrow mesenchymal stem cells.	[189]
Sensors	A chitosan-albumin cryogel with entrapped ferrocene and a layer of CNTs	Glucose biosensor	[152]
	A composite of chitosan, graphene, ionic liquid and ferrocene cryogel which was decorated with gold nanoparticles	Measuring prostate-specific antigen.	[190]
Bioreactors	Bacteria-based cryogels	Degradation of organic contaminates	[62,63]
	HEMA-alginate based cryogels	Bioartificial liver device	[191]
3D printing	Poly(n-hydroxyethyl acrylamide-co-methyl vinyl ketone)	Self-healing materials	[66]
	Carboxymethylcellulose based cryogels	A multilayer 3D printing of cryogels with local pore size change on demand	[64,65]

## Abbreviations

AAD	Adipic acid dihydrazide
AP	Apple pectin
APS	Ammonium persulphate
BDDE	Butanediol diglycidyl ether
bFGF	Basic fibroblasts growth factor
BM-MSC	Bone marrow mesenchymal stem cells
BMP-2	Bone morphogenic protein-2
CNT	Carbon nanotube
CpG-ODN	Cytosine-phosphodiester-guanine oligonucleotide
DMSO	Dimethyl sulfoxide
DNA	Deoxyribonucleic acid
DVS	Divinyl sulfone
ECM	Extracellular matrix
EDC	1-ethyl-3-(3-dimethylaminopropyl)carbodiimide
EGDE	Ethylene glycol diglycidyl ether
GA	Glutaraldehyde
GAG	Glucosaminoglycans
GDNF	Glial cell-derived neurotrophic growth factor
GlcN	Glucosamine
GM-CSF	Macrophage colony-stimulating factor
HA	Hyaluronic acid
HAP	Hydroxyapatite
HBMSC	Human bone marrow stromal cells
HEP	Heparin
HP	Heracleum pectin
HPLC	High-performance liquid chromatography
HUVEC	Human umbilical vein endothelial cells
MBA	N,N’-methylenebisacrylamide
MSC	Mesenchymal stromal cells
NGF	Nerve growth factor
nHAP	Nanohyroxyapatite
NHS	N-hydroxysuccinimide
NIPA	N-isopropylacrylamide
NIR	Near-infrared
NVP	N-vinyl-2-pyrrolidone
PAMAM	Polyamidoamine
PBS	Phosphate-buffered saline
PDA	Polydopamine
PDGF-BB	Platelet-derived growth factor-BB
PEG	Poly(ethylene glycol)
PEGDA	Poly(ethylene glycol) diacrylate
PEGDE	Poly(ethylene glycol) diglycidyl ether
PEO	Poly(ethylene oxide)
pHEMA	Poly(hydroxyethylmethacrylate)
PL	Platelet lysate
PLGA	Poly(lactic-co-glycolic acid)
PLLA	Poly(L-lactic acid)
PVA	Poly(vinyl alcohol)
PVC	Poly(vinyl caprolactam)
PVP	Polyvinylpyrrolidone
rBMSCs	Rabbit bone marrow-derived stem cells
ROS	Reactive oxygen species
SF	Silk fibroin
STF	Simulated tear fluid media
TEMED	Tetramethylethylenediamine
TGase	Transglutaminase
VEGF	Vascular endothelial growth factor
